# Identification of clinically achievable combination therapies in childhood rhabdomyosarcoma

**DOI:** 10.1007/s00280-016-3077-8

**Published:** 2016-06-20

**Authors:** Elliot Kahen, Diana Yu, Douglas J. Harrison, Justine Clark, Pooja Hingorani, Christopher L. Cubitt, Damon R. Reed

**Affiliations:** 1 Sunshine Project Translational Research Lab, H. Lee Moffitt Cancer Center and Research Institute, Tampa, FL USA; 2 Chemical Biology and Molecular Medicine Program, H. Lee Moffitt Cancer Center and Research Institute, Tampa, FL USA; 3 Sarcoma Department, H. Lee Moffitt Cancer Center and Research Institute, Tampa, FL USA; 4 Adolescent and Young Adult Program, H. Lee Moffitt Cancer Center and Research Institute, 12902 Magnolia Drive, 33612 Tampa, FL USA; 5 Division of Pediatrics, MD Anderson Cancer Center, Houston, TX USA; 6 Center for Cancer and Blood Disorders, Phoenix Children’s Hospital, Phoenix, AZ USA

**Keywords:** Rhabdomyosarcoma, Combination chemotherapy, Clinically achievable concentrations, AZD1775, Cyclophosphamide, Etoposide, Irinotecan

## Abstract

**Purpose:**

Systemic therapy has improved rhabdomyosarcoma event-free and overall survival; however, approximately 40 % of patients will have progressive or recurrent disease which is difficult to cure and remains a considerable challenge. Minimal progress has been made in improving outcomes for metastatic or relapsed RMS due to a lack of effective therapeutic agents. Targeted therapies are likely to be incorporated into regimens which rely on conventional cytotoxic chemotherapy. A system to evaluate novel combinations of interest is needed.

**Methods:**

In this study, we explored 8 agents, 5 that are routinely used or similar to agents used in the clinical management of RMS and 3 biologically targeted agents with novel mechanisms of action, the Wee1 inhibitor AZD1775, the tyrosine kinase inhibitor cabozantinib, and the proteasome inhibitor bortezomib. All were tested individually at clinically achievable concentrations for activity in 4 RMS cell lines and then for potential synergy in two-drug combinations.

**Results:**

We found single-agent activity in five of the agents (or their active metabolites) that constitute the standard of care in RMS and for AZD1775 with mean IC50 values of 207 ng/ml, well below clinically achievable levels. In addition, the combination of individual cytotoxic chemotherapeutics currently used for RMS demonstrated largely synergistic activity with higher, but clinically achievable concentrations of AZD1775 in our assays.

**Conclusions:**

Prioritization of chemotherapeutics in RMS is possible using an in vitro system that can define novel drug combinations worthy of future investigation. AZD1775 exhibits single-agent activity, as well as synergy with conventional cytotoxic chemotherapy, and is a novel targeted agent that warrants further study in RMS.

**Electronic supplementary material:**

The online version of this article (doi:10.1007/s00280-016-3077-8) contains supplementary material, which is available to authorized users.

## Introduction

Rhabdomyosarcoma (RMS) is the most common soft-tissue sarcoma (STS) in children and young adults with approximately 350 patients diagnosed each year in the USA [1]. Treatment is multimodal and includes systemic chemotherapy and local control of bulk disease which usually employs radiation therapy and in certain cases, surgical resection. Outcomes for low- and intermediate-risk RMS (typically stratified by stage, surgical resectability, and histology) remain favorable with overall survival of 98 and 79 %, respectively. Patients with high-risk disease, as defined by the presence of metastasis, continue to have poor outcomes with an overall survival of 56 %, with EFS as low as 21 % in certain cases depending on the presence of other known poor prognostic indicators [2–9].

Recent strategies to improve outcome for high-risk patients have included intensification of systemic chemotherapy with interval compression of alkylating agents. This approach, evaluated in the Children’s Oncology Group Study ARST0431, led to no significant benefit in outcome, as compared to prior studies, with a 3-year event-free survival of 38 % [10]. Novel treatment strategies directed against molecular targets are needed in this patient population especially as conventional treatment strategies that rely on cytotoxic chemotherapeutics are limited [11].

Several agents have been developed which target novel and previously unexplored molecular pathways in RMS while also demonstrating preclinical activity. Cabozantinib (XL184) is a tyrosine kinase inhibitor of the c-MET and RET kinase pathway as well as the vascular endothelial growth factor receptor 2 (VEGFR2) which impairs tumor cell proliferation and angiogenesis [12–16]. In both alveolar and embryonal RMS, MET signaling has been found to impede myogenic differentiation, promote tumor cell proliferation and growth, and increase metastatic potential [14, 15]. The proteasome inhibitor, bortezomib, functions through inhibition of the 26S proteasome, leading to apoptosis, cell cycle arrest, and deregulated NF-KB signaling and is currently approved for use in hematologic malignancies [17–20]. In vitro studies have shown RMS cell lines to exhibit increased rates of apoptosis and cell cycle arrest when treated with bortezomib both as a single agent as well as in combination [19, 20]. AZD1775 is a selective tyrosine kinase inhibitor of the Wee1 kinase which regulates the cell cycle through phosphorylation and inhibition of cyclin-dependent kinase 1. The agent has been found to inhibit the growth of several sarcoma cell lines of varying histology. Furthermore, in osteosarcoma cell lines and patient-derived osteosarcoma murine xenografts, the combination of gemcitabine and AZD1775 was found to demonstrate significant synergistic activity. In RMS, the role of the Wee1 kinase is not yet known [21–26].

We screened 8 drugs as single agents (5 agents known to be active in RMS—4HC, an active metabolite of cyclophosphamide, SN-38, the active metabolite of irinotecan, etoposide, dactinomycin, and vinorelbine (microtubule inhibitor similar to vincristine) and 3 novel agents of interest—cabozantinib, bortezomib, and AZD1775) and in two-drug combinations using an automated screening method developed in our laboratory [27]. The methodology was optimized to incorporate clinically achievable drug concentrations and lengths of exposure that are possible based on human pharmacokinetic data [28]. By using drugs under evaluation in active and recently completed pediatric trials and agents with preclinical data documenting activity in RMS, we anticipate that we could efficiently develop strong preclinical data to help inform additional preclinical work and eventually clinical trials in RMS. The overall goal of this study was to identify combinations that exhibit in vitro activity while maintaining synergy and have the potential to be studied further in the context of in vivo and clinical evaluations.

## Materials and methods

### Investigational agents

Agents used included both cytotoxic and targeted agents (see Supplemental Table S2 for the vendor and catalog number). Stock solutions were made for each compound in DMSO at 4000× of the highest concentrations used in experiments. Chemical structures for all agents are publicly available.

### Cell culture

We selected two embryonal RMS (ERMS) (RD and SMS-CTR) and two ARMS cell lines (RH30 and RH41) that are well characterized and commonly used in recent studies [29]. ERMS cell lines, RD and SMS-CTR, were a gift from Dr. Calvin K. Lee at H. Lee Moffitt Cancer Center. ARMS lines, RH30 and RH41, were obtained from the Children’s Oncology Group Cell Line and Xenograft Repository (Texas, USA). Cells were maintained in RPMI with 10 % FBS. Cells were grown at 37 °C and 5 % CO_2_. All cell lines tested free of mycoplasma with MycoAlert tests (Lonza Rockland, Rockland, ME). Cell line identity was confirmed using StemElite ID system (Promega, Madison, WI) using the manufacturer’s instructions and the ATCC STR profile database.

### Single-agent screening

**Fig. 1:**
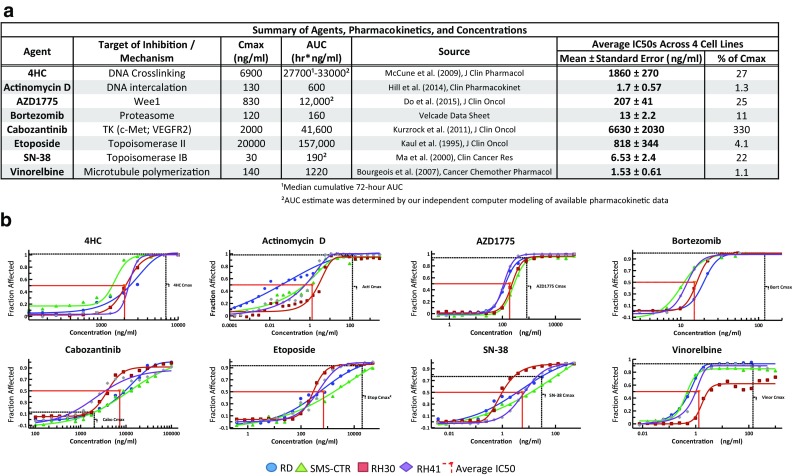
Single-agent activities of 8 therapeutic compounds screened against 4 RMS cell lines. **a** PK and IC50 values of the 8 drug candidates for the 4 RMS lines and comparison with clinically derived in vivo Cmax values. **b** Full dose–response curves of the drug candidates demonstrating single-agent cytotoxicity in the 4 RMS cell lines. The *red lines* indicate the in vitro IC50 levels and the *black lines* indicate the serum Cmax of each drug

Human pharmacokinetic data were collected for all agents from previously reported phase I studies, using pediatric and combination studies when available (Fig. 1a). Single-agent activities of a panel of 8 therapeutic candidates were characterized with 4 pediatric RMS cell lines (RD, SMS-CTR, RH30, and RH41). Dose–response curves were obtained for each drug in the panel, and single-agent anti-tumor activities were assessed using CellTiter-Glo luminescence cell viability assay at 72 h following drug application, a time which was optimized based on cell line growth characteristics. The fraction affected (FA) was calculated as FA = 1 − (CT-glo signal with drug treatment)/(CT-glo signal without drug treatment). This reflects the reduction in the cellular metabolism (a correlate of cell viability) due to the drug treatment.

### Two-drug combination screening

A 5 × 5 checker-board matrix format was used to assess all two-drug combinations at five clinically achievable concentrations. Notably, these concentrations were selected at clinically achievable ranges along a range that allowed for less than optimal effect on cells in order to detect synergy. A full description of the methods was published previously [27]. Briefly, each combination was evaluated at multiple drug ratios to identify synergy (Supplemental Table S2). In cases where the same dilution factors were used for both drugs of the combination, diagonals of the 5 × 5 checker-board matrix provide the effects of the drug combination at constant drug ratio. Full dose–response curves were obtained for each individual drug, and the combination index (CI) for all combinations was calculated using CalcuSyn 2.0 and custom-designed analysis package based on the Chou-Talalay method.

### Cell viability assays

The activity levels of single agents and combinations were determined by a high-throughput CellTiter-Glo cell viability assay (Promega). Cells (1–2 × 10^3^) were plated in each well of 384-well plates using a Precision XS liquid handling station (Bio-Tek Instruments, Winooski, VT) and incubated overnight. Drug source plates were prepared in 96-well Megatiter plates (Neptune Scientific, San Diego, CA), and the Precision XS station was used to transfer drugs to four replicate wells with an additional four control wells receiving DMSO vehicle control without drug. At the end of the drug incubation period, CellTiter-Glo or Caspase-Glo reagent was added to each well at 1:1 ratio (v/v) with media. The luminescence of the product of viable cells was measured with a Synergy 4 microplate reader (Bio-Tek Instruments). The luminescence data were transferred to Microsoft Excel to calculate percent viability. IC50 values were determined using a sigmoidal equilibrium model regression and XLfit version 5.2 (ID Business Solutions). The IC50 values obtained from single-drug cell viability assays were used to design subsequent drug combination experiments. High-throughput two-agent combination screening experiments were performed using a 5 × 5 matrix format in 384-well plates to interrogate 25 individual concentration ratios per combination.

### Analysis of additive and synergistic effects in combination screening data

For drug combination experiments, the CellTiter-Glo assay was used to measure cell viability, with results analyzed for synergistic, additive, or antagonistic effects using primarily the combination index (CI) method of Chou-Talalay [30] with additional supporting analysis from fold of potentiation (FOP). For the CI method, the dose–effect curve for each drug was determined based on experimental observations using the median-effect principle and was compared to the effect achieved with the two-drug combination to derive a CI value. This method involves plotting dose–effect curves for each single agent using the median-effect equation: fa/fu = (*D*/*D*_m_)_m_, where *D* = dose of the drug, *D*m = dose required for 50 % effect, fa and fu = affected and unaffected fractions, respectively (fa = 1 − fu), and *m* = exponent signifying the sigmoidicity of the dose–effect curve. XLfit computer software was used to calculate *D*_m_ and *m*. CIs used for the analysis of the drug combinations were determined by the isobologram equation for mutually nonexclusive drugs that have different modes of action: CI = (*D*)_1_/(*D*_x_)_1_ + (*D*)_2_/(*D*_x_)_2_ + (*D*)_1_(*D*)_2_/(*D*_x_)_1_(*D*_x_)_2_, where (*D*_x_)_1_ and (*D*_x_)_2_ in the denominators are the doses (or concentrations) for *D*_1_ (Drug1) and *D*_2_ (Drug2) alone that gives *x* % inhibition, whereas (*D*)_1_ and (*D*)_2_ in the numerators are the doses of Drug1 and Drug2 in combination that also inhibited *x* % (i.e., isoeffective). CI calculations were done in custom Microsoft Excel templates and verified with CalcuSyn 2.0 (Biosoft, Cambridge, UK). CI < 1, CI = 1, and CI > 1 indicate synergism, additive effects, and antagonism, respectively.

Fold-of-potentiation (FOP) analysis was used for combination screening data with non-constant molar ratios to demonstrate the enhancement of one drug’s effect by another by measuring shift in IC50 [31, 32]. Curve fitting for FOP was performed using Prism v6.05 (GraphPad Software, La Jolla, CA, www.graphpad.com). Dose–response plots for single agents and drug combinations were fitted using a four-parameter nonlinear least-squares regression model. Curves were extrapolated to relevant maximum and minimum response levels.

### Cluster analysis

Prior to clustering, the FA and CI data were normalized using the Kahen-Yu method: Data are log-transformed and converted to a common scale by multiplying the log-transformed 1-FA by a coefficient of 1/3. Both variables were then multiplied by −10. This Kahen-Yu transformation results in FA and CI values that are suitable for concurrent input into the subsequent cluster analysis. Cluster analysis was accomplished with the use of Cluster 3.0 (Stanford University Labs, Stanford, CA). Complete-linkage unsupervised hierarchical clustering of FA and CI values together was performed using uncentered absolute correlation similarity metrics. Java TreeView 1.1.6r4 (Stanford University Labs) was employed to visualize clustered data.

## Results

### Single-agent activity against ERMS and ARMS cell lines

We first characterized the single-agent activity of a panel of 8 therapeutic candidates (Fig. 1a) using 2 ERMS cell lines (RD and SMS-CTR) and 2 ARMS cell lines (RH30 and RH41). Of the 8 drugs in the panel, 5 agents (vinorelbine, SN-38, 4HC, etoposide, and actinomycin) were included for their known therapeutic activity in RMS treatment and 3 agents (cabozantinib, bortezomib, and AZD1775) were chosen for evidence of either a rational therapeutic target or preclinical activity in sarcomas. We generated the dose–response curves for each drug on each cell line to assess the sensitivity of the RMS cells to the compounds (Fig. 1c, Supplemental Fig. 1). CellTiter-Glo luminescence assay was used to assess the anti-tumor activities by measuring the ATP levels, an indicator of cellular metabolism, at 72 h post-drug application. We calculated the IC50s from the dose–response curves and compared them to previously reported serum Cmax levels in clinical studies as a preliminary indicator of the feasibility of these drugs for RMS in the clinical setting (Fig. 1a). Of the 8 drugs we tested, with the exception of cabozantinib, all had IC50s well below the reported serum Cmax, demonstrating efficacy against RMS cells at clinically achievable levels (Fig. 1b). Despite the differences in the genetic translocations and clinical presentations that characterize ERMS and ARMS, we did not see a significant difference in the sensitivity of the two types of RMS cells to the majority of the drugs in our panel (Fig. 1b).

### Evaluation for active and synergistic combinations

**Fig. 2:**
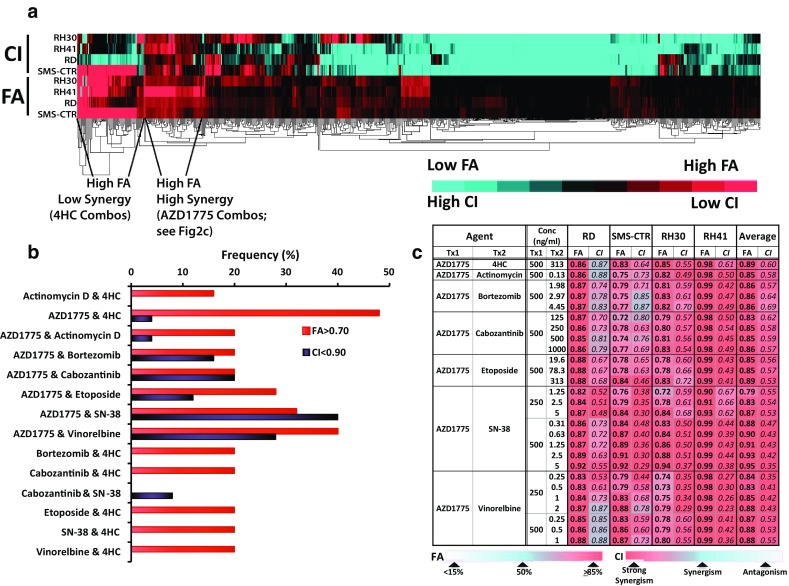
Combination screening results. **a** Clustering results showing top combination picks based on FA. **b** Frequency plot showing the combinations that demonstrated good efficacy (FA > 0.75) as well as synergy (CI < 0.7) and/or additivity (CI < 1.1). **c** FA and CI values for the top combinations selected using the clustering technique

Following characterization of the single-agent anti-tumor activities, we used a 5 x 5 checker-board matrix format to assess two-drug combinations of the 8 chemotherapy agents at 5 clinically achievable concentrations and 9–25 different drug ratios to evaluate anti-tumor activity and identify synergy (Supplemental Table S2). The fraction of cell population sensitive to each drug combination (FA) was assessed using CellTiter-Glo luminescence assay, and the combination index (CI) values for the 28 two-drug combinations were calculated using CalcuSyn 2.0 and custom-designed analysis package based on the Chou-Talalay method (additional details of the method were published in Yu et al. 2015). We performed clustering analysis on our screening data using the FA and CI attributes to highlight drug combinations that could potentially be promising for the treatment of pediatric RMS (Fig. 2a). The results from our combination screening indicate that multiple drug combinations involving AZD1775 and 4HC produced high FA values and thus were very effective in eliminating the RMS cells. However, while a number of 4HC drug combinations produced >95 % anti-tumor activities (4HC:bortezomib, 4HC:cabozantinib, and 4HC:SN-38), they also had CI > 1.1, indicating antagonism in the drug pairs. In contrast, several AZD1775 drug combinations demonstrated both high FA and low CI values, indicating good anti-tumor activity against the RMS cells and synergy within the drug pairs (Fig. 2a). Since each combination was assessed at multiple drug levels and drug ratios, we used a frequency plot to indicate the percentage of the drug pairs that produced good effect levels (at FA > 0.70), as well as demonstrated synergy (CI < 0.9) (Fig. 2b) and at lower thresholds of activity (FA > 0.5 and CI < 1.1, Supplemental Fig. 2). In addition, FA and CI values of these combinations in all 4 RMS cell lines are summarized in Fig. 2c (complete screening results are provided in Supplemental Table S1).

### Combination of AZD1775 with alkylating agent, 4HC

**Fig. 3:**
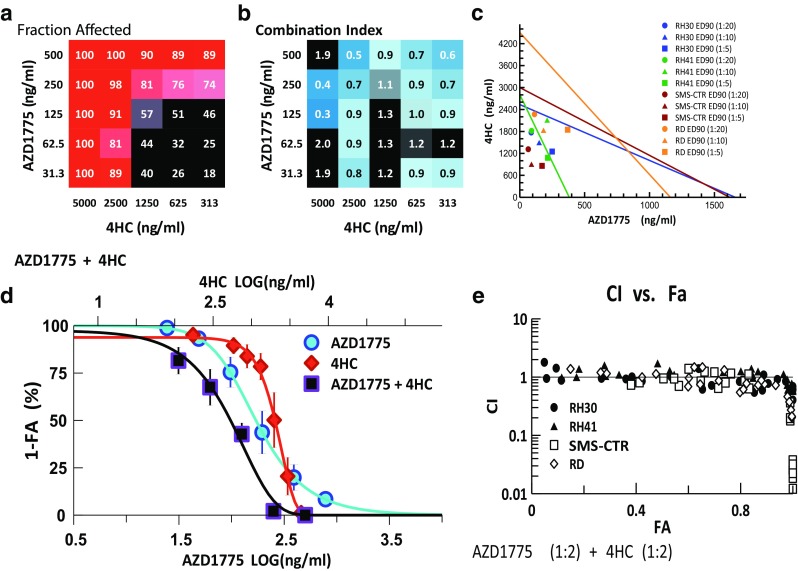
Combination activity for AZD1775 and 4HC. **a** FA and **b** CI values of AZD1775 and 4HC assessed at 25 different concentrations. **c** Isobologram analysis of the combination demonstrating synergy at ED90 with 3 different drug ratios. **d** Single and combination drug–response curves for the combination showing cytotoxicity of the drug combination compared to single agent. **e** CI–FA plot showing similar responses in all 4 RMS cells lines to this drug combination

Combination of 2500 ng/ml 4HC with varying levels of AZD1775 (32–500 ng/ml) produced FA values between 90 and 100 % (Fig. 3a) while demonstrating synergy (CI < 0.9) (Fig. 3b). Isobolograms at the ED90 level, the concentrations necessary to reduce the measured viability by 90 % in a given cell line at a given drug ratio, of the combination at 1:20, 1:10, and 1:5 drug ratios (AZD1775:4HC) confirm the synergistic interactions between the two drugs (Fig. 3c). This synergy results in a leftward shift in the dose–effect curve of the combination and a respective 1.8- and 2.0-fold reduction in the IC50s of AZD1775 and 4HC in the combination compared to that of single agent alone (Fig. 3d). The CI–FA plot further demonstrates that synergy is observed at higher FA and the robustness of this response in the 4 RMS cell lines used (Fig. 3e).

### Combination of AZD1775 with topoisomerase inhibitors SN-38 and etoposide

**Fig. 4:**
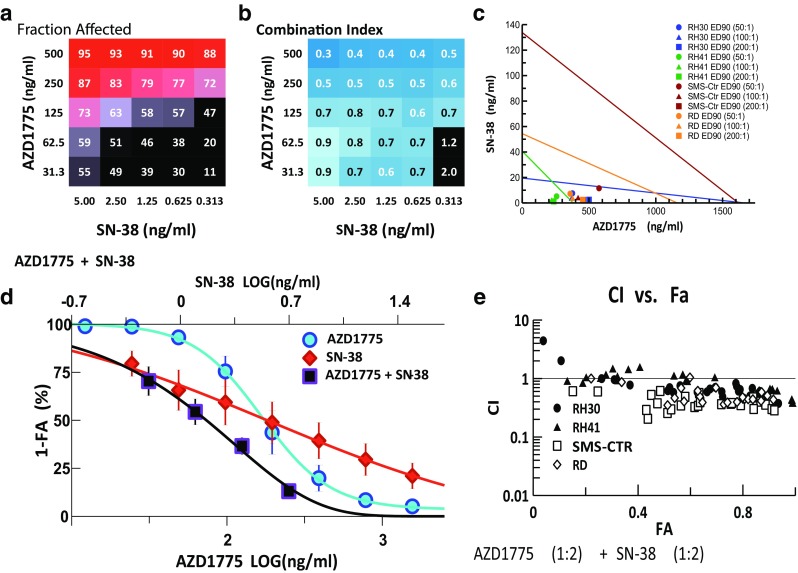
Combination activity for AZD1775 and SN-38. **a** FA and **b** CI values of AZD1775 and SN-38 assessed at 25 different concentrations. **c** Isobologram analysis of the combination demonstrating synergy at ED90 with 3 different drug ratios. **d** Single and combination drug–response curves for the combination showing cytotoxicity of the drug combination compared to single agent. **e** CI–FA plot showing synergy in all 4 RMS cells lines across a broad arrange of FA values in this drug combination

**Fig. 5:**
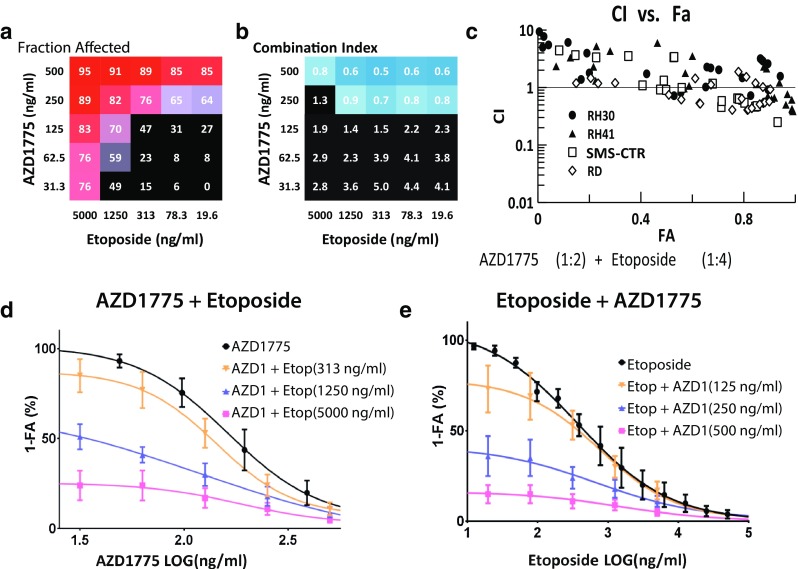
Combination activity for AZD1775 and etoposide. **a** FA and **b** CI values of AZD1775 and etoposide assessed at 25 different concentrations. **c** CI–FA plot showing synergy at higher FA values. **d** Fold-of-potentiation graph demonstrating the increase in cytotoxic activity of adding etoposide at 313, 1250, and 5000 ng/ml to varying concentrations of AZD1775. **e** Fold-of-potentiation graph demonstrating the increase in cytotoxic activity of adding AZD1775 at 125, 250, and 500 ng/ml to varying concentrations of etoposide

Combinations of AZD1775 with the two topoisomerase inhibitors on our panel (etoposide and SN-38, the active metabolite of irinotecan) produced similar results in cytotoxicity and synergy, potentially pointing to the robustness of a combination with these two types of agents against RMS cells (Figs. 4, 5). In both instances, the best results were achieved when AZD1775, at 250 and 500 ng/ml, was combined with varying concentrations of the latter (Figs. 4a, 5a). However, the combination of AZD1775 with SN-38 demonstrated synergy across a broader range of drug ratios and FA values. AZD1775 and SN-38 were cytotoxic against 70–95 % of RMS cells with CI values ranging from 0.3 to 0.9 and produced a respective 2.4- and 2.3-fold reduction in the IC50s of AZD1775 and SN-38 in the combination compared to that of single agent alone (Fig. 4b, d). Isobolograms at the ED90 level for 50:1, 100:1, and 200:1 (AZD1775:SN28) drug ratios confirm the synergistic interactions between the two drugs (Fig. 4c). Furthermore, the CI–FA plot demonstrates that synergy is observed throughout the range of FA values, with slight increases in synergy at the higher FA values (Fig. 4e). Etoposide and AZD1775 fold-of-potentiation graphs also demonstrated increased cytotoxicity in combination (Fig. 5d).

### Combination of AZD1775 with microtubule inhibitor, vinorelbine

**Fig. 6:**
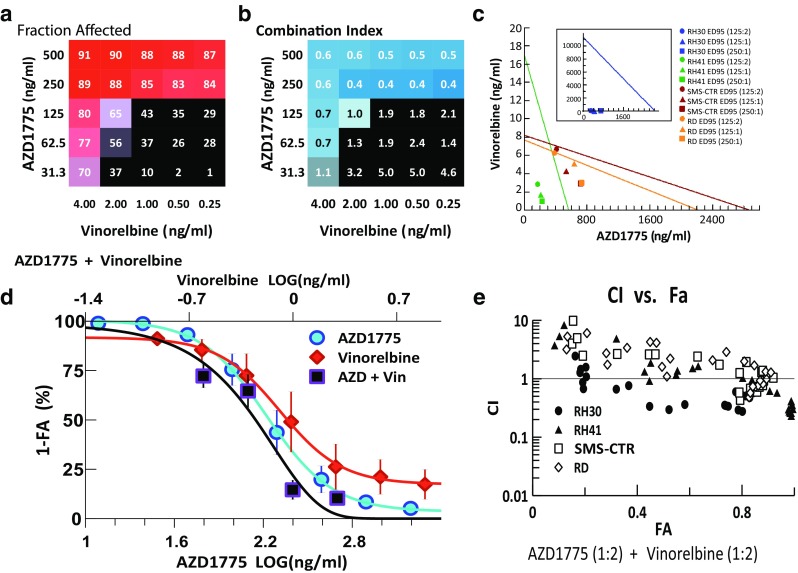
Combination activity for AZD1775 and vinorelbine. **a** FA and **b** CI values of AZD1775 and vinorelbine assessed at 25 different concentrations. **c** Isobologram analysis of the combination demonstrating at ED90 with 3 different drug ratios. **d** Single and combination drug–response curves for the combination showing cytotoxicity of the drug combination compared to single agent. **e** CI–FA plot showing slight differential responses in the 2 ARMS cells lines to this drug combination

Finally, synergy was also detected when combining AZD1775 with vinorelbine, an inhibitor of microtubule assembly, where the combination demonstrated strong synergy (CI < 0.6) and high efficacy against the RMS cells (FA range 83–91 %) (Fig. 6a, b). Isobolograms of the combination at the ED95 level showed synergy for drug ratios at 125:1, 125:2, and 250:1 (AZD1775:vinorelbine) (Fig. 6c). The dose–effect curve for the combination demonstrated a 1.2- and 3.0-fold reduction in the IC50s of AZD1775 and vinorelbine, respectively (Fig. 6d). Interestingly, the CI–FA plot for this combination showed a difference in the response of the two ARMS lines, where RH30 demonstrated synergy across a broader range of FA values than the other 3 cell lines (Fig. 6e). However, this combination was synergistic in all RMS lines at FA > 0.8.

## Discussion

Our results confirm that RMS cell lines are sensitive to multiple cytotoxic agents commonly used in frontline therapy including cyclophosphamide, dactinomycin, etoposide, irinotecan, and vinorelbine. We also found that cabozantinib, bortezomib, and AZD1775 exhibit robust activity in vitro both alone and in combination similar to conventional agents.

The most robust combinations as defined by producing a high FA value while maintaining a synergistic CI included the Wee1 kinase inhibitor, AZD1775. Combinations that employed a proteasome inhibitor, bortezomib, and a tyrosine kinase inhibitor, cabozantinib, also demonstrated activity by FA measurements, but with CI > 1.1 indicating antagonism with standard agents.

Both the mechanism of AZD1775 and a review of the literature support its use as an agent in combination with conventional chemotherapy in RMS, which may explain its high cell kill as measured by FA while maintaining synergy. The Wee1 kinase maintains cells in G2/M arrest, providing time for DNA repair prior to mitosis. When the Wee1 kinase is inhibited, CDK1/2 activity proceeds unchecked, and cells progress prematurely through G2/M leading to mitotic progression, DNA strand breaks, mitotic catastrophe, and cell death [21, 22]. In addition to growth inhibition of several sarcoma cell lines, AZD1775 in combination with gemcitabine demonstrated synergistic activity in osteosarcoma cell lines and patient-derived osteosarcoma murine xenografts. Specifically, the agents led to a more significant delay in tumor growth and smaller tumors in combination than when either agent was given alone [23]. It is postulated that the combination of this agent with conventional chemotherapy potentiates the DNA damage exerted by standard cytotoxics by impeding this critical cell cycle checkpoint [33]. Certainly, our data confirm the activity of AZD1775 in combination with alkylating agents, topoisomerase inhibitors, and microtubule inhibitors in RMS in vitro. In RMS, the impact of Wee1 kinase inhibition is not yet known though the completion of a phase I trial demonstrating good tolerance will allow for more robust trials with this agent [25, 26].

Based on the promising in vitro data, there is strong interest in further development of AZD1775 in the pediatric setting. The Children’s Oncology Group (COG) is currently studying the agent in two phase I trials—one that is evaluating the agent in combination with radiation therapy for patients with newly diagnosed diffuse intrinsic pontine glioma (NCT01922076) and one that is evaluating the agent in combination with irinotecan in patients with refractory solid tumors (NCT02095132). There remains high interest in this agent for patients with RMS as well. Evaluating the addition of AZD1775 to conventional chemotherapy is consistent with the overall strategy of the COG’s Soft Tissue Sarcoma Committee to conduct randomized phase II studies in patients with metastatic rhabdomyosarcoma to identify agents worthy of larger phase III studies.

Despite the promising results seen in this study with AZD1775 especially in combination with 4HC, SN-38, and vinorelbine, several limitations exist. We recognize that for some agents, serum levels may over-represent agent delivery to tumor cells or in other situations may underestimate agent delivery based on either conditions in the tumor or the microenvironment. Unfortunately, intratumoral concentrations of many agents are not available. We also recognize that not all agent effects can be determined in vitro with stromal, vascular, and immunologic activities being some examples of activities not represented with our system. For example, it is possible that our observation of low cabozantinib activity is due to the inability of the cell culture model to fully recapitulate the complex RMS tumor microenvironment in vivo, and thus is not able to fully assess the potency of this drug. Importantly, we acknowledge that this system is intended to explore a number of agents and combinations that could not reasonably be investigated in patients or animal models and acknowledge it is yet to be proven that these methods of incorporating clinically achievable concentrations will be more informative for clinical translation than prior in vitro studies that do not consider human pharmacokinetic data. While activity was maintained across both ARMS and ERMS cell lines, the results, both alone and in combination, should ultimately be confirmed in other models including possibly an in vivo model or a clinical trial in patients with RMS.

Despite the limitations of the system, our data provide support for further development of AZD1775 in RMS, especially in combination with cytotoxic chemotherapy going forward and especially for patients with high-risk or recurrent disease who have a dismal prognosis that has not improved over several decades despite multiple clinical trials. Furthermore, this in vitro system represents an efficient method to rapidly screen novel agents in combination and prioritize combinations that should be considered for additional evaluation in rare diseases such as RMS.

## Electronic supplementary material

Below is the link to the electronic supplementary material.

Supplementary material 1 (PDF 1368 kb)
