# Supplementary material for: Identification of clinically achievable combination therapies in childhood rhabdomyosarcoma
Source: Cancer Chemother Pharmacol. 2016 Jun 20;78:313–23. doi: 10.1007/s00280-016-3077-8 (PMC4965487; doi:10.1007/s00280-016-3077-8)
Supplement: Supplementary file 1 — Supplementary material 1 (PDF 1368 kb) [file 280_2016_3077_MOESM1_ESM.pdf]

Identification of Clinically Achievable Combination Therapies in Childhood Rhabdomyosarcoma – Supplementary Figures

Elliot Kahen[1*], Diana Yu [1*], Douglas Harrison[2], Justine Clark[1], Pooja Hingorani[3], Christopher Cubitt[1,3] and Damon Reed[1,4,5,6]

*equal contribution

[1]Sunshine Project and Translational Research Lab, [4]Chemical Biology and Molecular Medicine Program, [5]Sarcoma Department, [6]Adolescent and Young Adult Program; H. Lee Moffitt Cancer Center and Research Institute, Tampa, Florida; [2]Division of Pediatrics, MD Anderson Cancer Center, Houston, TX; [3]Center for Cancer and Blood Disorders, Phoenix Children's Hospital, Phoenix, Arizona

Corresponding author: damon.reed@moffitt.org

Supplemental Fig 1: Fraction affected values for single agent dosage response

4HC

Conc (ng/ml)	Cell Line			
	RD	SMS-CTR	RH30	RH41
10000.00	1.000	1.000	1.000	1.000
6666.67	1.000	1.000	1.000	1.000
4444.44	0.993	0.964	1.000	1.000
2962.96	0.518	0.972	0.791	0.898
1975.31	0.377	0.915	0.457	0.244
1316.87	0.321	0.344	0.147	0.050
877.91	0.225	0.305	0.054	0.061
585.28	0.099	0.230	0.028	0.063
390.18	0.033	0.178	0.012	0.033
260.12	0.018	0.168	0.029	0.022
173.42	0.059	0.120	0.021	-0.005
115.61	0.065	0.113	0.025	0.033

Etoposide

Conc (ng/ml)	Cell Line			
	RD	SMS-CTR	RH30	RH41
50000.00	0.981	0.892	0.979	0.996
25000.00	0.958	0.860	0.969	0.986
12500.00	0.914	0.760	0.956	0.971
6250.00	0.845	0.666	0.953	0.951
3125.00	0.762	0.587	0.933	0.917
1562.50	0.634	0.422	0.899	0.861
781.25	0.492	0.334	0.815	0.694
390.63	0.449	0.392	0.648	0.389
195.31	0.446	0.305	0.339	0.196
97.66	0.386	0.297	0.135	0.319
48.83	0.167	0.145	0.044	0.142
24.41	0.073	-0.007	0.037	0.126
12.21	0.026	-0.010	0.057	0.061
6.10	0.002	0.089	0.051	0.030
3.05	-0.022	0.034	0.044	0.025
1.53	0.006	-0.056	0.045	-0.007
7.63E-01	0.013	-0.015	0.030	-0.026
3.81E-01	-0.010	-0.062	0.042	0.005

Actinomycin D

Conc (ng/ml)	Cell Line			
	RD	SMS-CTR	RH30	RH41
3000.00	0.987	0.962	0.921	0.992
1500.00	0.988	0.963	0.934	0.993
750.00	0.987	0.956	0.930	0.993
375.00	0.984	0.934	0.916	0.992
187.50	0.981	0.913	0.912	0.995
93.75	0.984	0.846	0.949	0.998
46.88	0.993	0.954	0.982	0.999
23.44	0.997	0.931	0.997	1.000
11.72	0.998	0.971	0.967	0.999
5.86	0.979	0.921	0.783	0.988
2.93	0.751	0.763	0.387	0.892
1.46	0.682	0.537	0.327	0.644
7.32E-01	0.654	0.412	0.243	0.417
3.66E-01	0.625	0.353	0.221	0.323
1.83E-01	0.581	0.366	0.188	0.251
9.16E-02	0.575	0.384	0.165	0.261
4.58E-02	0.508	0.339	0.152	0.223
2.29E-02	0.446	0.340	0.204	0.253
1.14E-02	0.555		0.197	0.271
5.72E-03	0.438		0.087	0.198
2.86E-03	0.311		0.009	0.106
1.43E-03	0.226		0.011	0.076
7.15E-04	0.205		-0.018	0.081
3.58E-04	0.159		0.003	0.016
1.79E-04	0.130		-0.006	0.011
8.94E-05	0.113		0.001	0.025
4.47E-05	0.052		0.002	0.049

Bortezomib

Conc (ng/ml)	Cell Line			
	RD	SMS-CTR	RH30	RH41
50.00	0.982	0.947	0.993	0.980
33.33	0.928	0.922	0.985	0.961
22.22	0.646	0.873	0.860	0.897
14.81	0.204	0.762	0.392	0.723
9.88	0.061	0.413	0.041	0.269
6.58	0.029	0.062	0.016	-0.017
4.39	0.015		0.012	-0.031
2.93	0.003	0.009	0.012	-0.012

AZD1775

Conc (ng/ml)	Cell Line			
	RD	SMS-CTR	RH30	RH41
50000.00	0.987	0.963	0.994	1.000
25000.00	0.960	0.921	0.989	0.988
12500.00	0.965	0.930	0.963	0.992
6250.00	0.965	0.960	0.963	0.997
3125.00	0.957	0.953	0.956	0.997
1562.50	0.940	0.934	0.923	0.995
781.25	0.902	0.892	0.878	0.991
390.63	0.842	0.645	0.751	0.967
195.31	0.712	0.312	0.438	0.793
97.66	0.364	0.118	0.096	0.403
48.83	0.126	-0.001	0.012	0.136
24.41	0.046	-0.038	0.006	0.035
12.21	0.025	-0.004	0.014	0.006
6.10	0.026	-0.008	0.011	-0.006
3.05	0.023	-0.026	0.022	-0.006
1.53	-0.014	-0.041	0.019	-0.007
7.63E-01	0.033	-0.024	0.034	-0.032
3.81E-01	-0.012	-0.030	0.023	-0.021

SN-38

Conc (ng/ml)	Cell Line			
	RD	SMS-CTR	RH30	RH41
500.00	0.992	0.979	0.990	0.998
250.00	0.969	0.907	0.986	0.991
125.00	0.943	0.798	0.985	0.947
62.50	0.875	0.692	0.966	0.896
31.25	0.760	0.626	0.947	0.826
15.63	0.657	0.522	0.922	0.717
7.81	0.550	0.438	0.877	0.560
3.91	0.491	0.370	0.822	0.363
1.95	0.416	0.295	0.731	0.184
9.77E-01	0.429	0.298	0.570	0.074
4.88E-01	0.352	0.240	0.193	0.034
2.44E-01	0.187	0.126	0.011	0.014
1.22E-01	0.045	0.025	0.007	-0.027
6.10E-02	0.035	0.014	0.020	-0.035
3.05E-02	0.022	0.000	0.017	-0.019
1.53E-02	-0.001	0.004	0.013	-0.019
7.63E-03	0.006	-0.040	0.005	-0.032
3.81E-03	0.048	-0.022	-0.005	-0.013

Cabozantinib

Conc (ng/ml)	Cell Line			
	RD	SMS-CTR	RH30	RH41
100000.00	0.999	0.936	0.985	0.916
66666.67	0.973	0.856	0.968	0.860
44444.44	0.951	0.754	0.935	0.795
29629.63	0.942	0.660	0.892	0.745
19753.09	0.731	0.582	0.854	0.687
13168.72	0.535	0.498	0.766	0.672
8779.15	0.472	0.499	0.831	0.727
5852.77	0.419	0.394	0.775	0.706
3901.84	0.250	0.260	0.454	0.633
2601.23	0.191	0.149	0.311	0.447
1734.15	0.086	0.075	0.124	0.308
1156.10	0.060	-0.002	0.080	0.158
770.73	0.012	-0.037	0.061	0.088
513.82	0.015	0.009	0.066	0.048
342.55	-0.001		0.059	0.040
228.37	-0.025	-0.021	0.061	0.020
152.24	-0.014	-0.091	0.059	-0.001
101.50	0.001	-0.095	0.041	-0.026

Vinorelbine

Conc (ng/ml)	Cell Line			
	RD	SMS-CTR	RH30	RH41
1000.00		0.821	0.723	
500.00		0.834	0.679	0.860
250.00		0.849	0.668	0.883
125.00	0.953	0.862	0.548	0.898
62.50	0.945	0.828	0.528	0.919
31.25	0.933	0.860	0.578	0.928
15.63	0.935	0.861	0.659	0.925
7.81	0.930	0.858	0.605	0.914
3.91	0.893	0.877	0.526	0.858
1.95	0.874	0.843	0.393	0.840
9.77E-01	0.708	0.759	0.089	0.484
4.88E-01	0.421	0.486	0.004	0.189
2.44E-01	0.233	0.231	0.013	0.103
1.22E-01	0.138	0.111	0.003	0.101
6.10E-02	0.070	0.075	0.003	0.065
3.05E-02	0.021	0.052	-0.001	0.034
1.53E-02	0.025	0.040	0.007	0.022
7.63E-03	-0.010	0.009	-0.001	-0.019

FA:	0	0.200	0.400	0.600	0.800	0.900	1.000

Supplemental Fig 2: Frequency graph showing all two-drug combinations that demonstrated FA>0.50 and CI<1.10

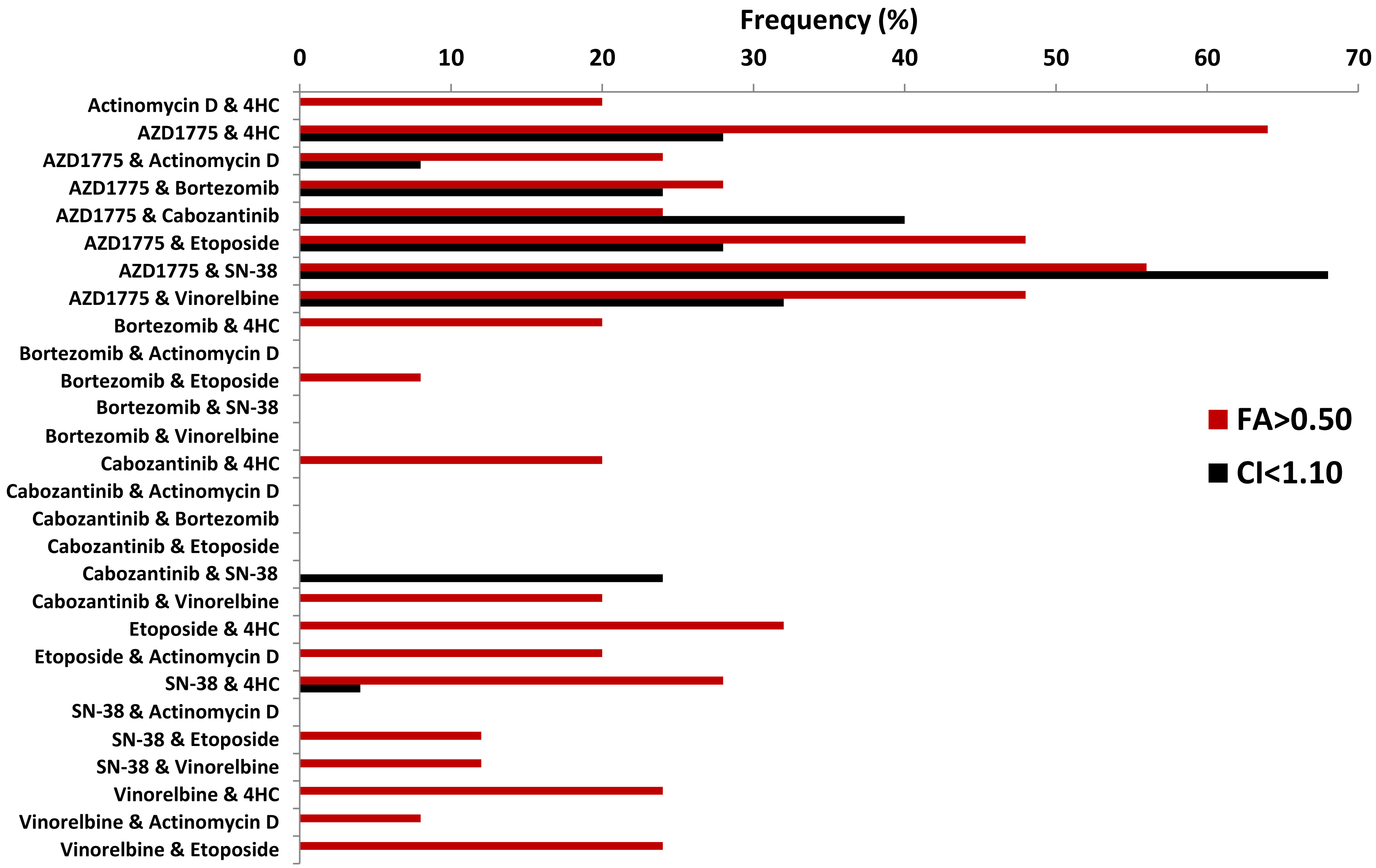

Agents Tx1	Agents Tx2	Mechanism Tx1	Mechanism Tx2	Conc (ng/ml) Tx1	Conc (ng/ml) Tx2	RD FA	RD CI	SMS-CTR FA	SMS-CTR CI	RH30 FA	RH30 CI	RH41 FA	RH41 CI	Average FA	Average CI
Actinomycin D	4HC	Alkylator	Alkylator	2	5000	0.893	3.94	0.999	0.10	0.999	0.95	0.998	1.02	1.00 ± 0.05	0.69 ± 1.68
					2500	0.650	5.00	0.998	0.07	0.381	3.00	0.452	4.93	0.61 ± 0.28	2.67 ± 2.31
					1250	0.614	5.00	0.491	5.00	0.212	5.00	0.359	5.00	0.35 ± 0.17	5.00 ± 0.00
					625	0.622	5.00	0.450	5.00	0.229	5.00	0.327	5.00	0.34 ± 0.17	5.00 ± 0.00
					313	0.642	5.00	0.417	5.00	0.178	5.00	0.314	5.00	0.30 ± 0.20	5.00 ± 0.00
				1	5000	0.836	4.22	0.999	0.09	0.999	0.96	0.998	1.01	1.00 ± 0.08	0.69 ± 1.82
					2500	0.596	5.00	0.998	0.07	0.501	1.73	0.364	3.66	0.62 ± 0.27	1.82 ± 2.16
					1250	0.552	5.00	0.429	5.00	0.202	4.17	0.193	4.78	0.27 ± 0.18	4.65 ± 0.39
					625	0.556	5.00	0.315	5.00	0.173	5.00	0.201	4.24	0.23 ± 0.17	4.75 ± 0.38
					313	0.573	5.00	0.298	5.00	0.166	5.00	0.169	4.61	0.21 ± 0.19	4.87 ± 0.20
				0.5	5000	0.796	3.90	0.999	0.10	0.999	0.95	0.998	0.98	1.00 ± 0.10	0.68 ± 1.66
					2500	0.489	5.00	0.997	0.08	0.534	1.50	0.214	3.38	0.58 ± 0.33	1.65 ± 2.15
					1250	0.443	5.00	0.402	5.00	0.129	5.00	0.069	5.00	0.20 ± 0.19	5.00 ± 0.00
					625	0.380	5.00	0.280	5.00	0.002	5.00	0.078	4.31	0.12 ± 0.18	4.77 ± 0.35
					313	0.404	5.00	0.247	5.00	0.013	5.00	0.054	5.00	0.10 ± 0.18	5.00 ± 0.00
				0.25	5000	0.726	3.98	0.998	0.11	0.999	0.95	0.996	1.05	1.00 ± 0.14	0.70 ± 1.69
					2500	0.426	5.00	0.998	0.06	0.329	1.84	0.145	2.89	0.49 ± 0.37	1.60 ± 2.06
					1250	0.291	5.00	0.398	3.83	0.072	5.00	0.033	4.45	0.17 ± 0.17	4.43 ± 0.56
					625	0.203	5.00	0.245	5.00	-0.017	5.00	0.042	3.41	0.09 ± 0.13	4.47 ± 0.79
					313	0.229	5.00	0.170	5.00	-0.051	5.00	0.023	4.66	0.05 ± 0.13	4.89 ± 0.17
				0.125	5000	0.697	3.64	0.999	0.10	0.998	1.00	0.998	0.96	1.00 ± 0.15	0.69 ± 1.53
					2500	0.379	5.00	0.997	0.08	0.495	1.45	0.101	2.57	0.53 ± 0.37	1.37 ± 2.08
					1250	0.211	5.00	0.406	2.93	0.011	5.00	0.019	3.62	0.15 ± 0.19	3.85 ± 1.04
					625	0.146	5.00	0.237	5.00	-0.001	5.00	0.054	1.72	0.10 ± 0.10	3.91 ± 1.64
					313	0.162	5.00	0.110	5.00	-0.027	5.00	-0.001	5.00	0.03 ± 0.09	5.00 ± 0.00
AZD1775	Actinomycin D	Wee1	Alkylator	500	2	0.861	3.38	0.749	1.04	0.788	0.64	0.960	1.22	0.83 ± 0.09	0.97 ± 1.23
					1	0.836	2.58	0.747	0.89	0.737	0.75	0.970	0.85	0.82 ± 0.11	0.83 ± 0.88
					0.5	0.854	1.47	0.776	0.70	0.727	0.75	0.977	0.65	0.83 ± 0.11	0.70 ± 0.39
					0.25	0.863	1.04	0.811	0.56	0.785	0.58	0.983	0.53	0.86 ± 0.09	0.56 ± 0.24
					0.125	0.863	0.88	0.752	0.73	0.820	0.49	0.983	0.50	0.85 ± 0.10	0.58 ± 0.19
				250	2	0.689	5.00	0.517	4.69	0.571	0.99	0.496	5.00	0.53 ± 0.09	3.56 ± 1.96
					1	0.642	5.00	0.493	3.32	0.510	1.01	0.359	5.00	0.45 ± 0.12	3.11 ± 1.89
					0.5	0.518	5.00	0.324	5.00	0.277	2.26	0.233	5.00	0.28 ± 0.13	4.09 ± 1.37
					0.25	0.357	5.00	0.390	2.91	0.232	2.31	0.183	5.00	0.27 ± 0.10	3.41 ± 1.40
					0.125	0.313	5.00	0.541	0.93	0.245	1.88	0.148	5.00	0.31 ± 0.17	2.60 ± 2.11
				125	2	0.638	5.00	0.356	5.00	0.509	0.97	0.535	3.92	0.47 ± 0.12	3.30 ± 1.91
					1	0.582	5.00	0.253	5.00	0.457	0.85	0.494	2.70	0.40 ± 0.14	2.85 ± 2.01
					0.5	0.470	5.00	0.228	5.00	0.248	1.86	0.537	1.70	0.34 ± 0.16	2.85 ± 1.86
					0.25	0.476	5.00	0.213	5.00	0.244	1.36	0.527	1.36	0.33 ± 0.16	2.57 ± 2.10
					0.125	0.485	3.20	0.262	4.65	0.274	0.93	0.515	1.21	0.35 ± 0.13	2.26 ± 1.75
				62.5	2	0.695	5.00	0.511	4.34	0.561	0.58	0.520	3.56	0.53 ± 0.09	2.83 ± 1.95
					1	0.627	5.00	0.418	5.00	0.512	0.48	0.372	2.93	0.43 ± 0.11	2.80 ± 2.15
					0.5	0.505	5.00	0.327	5.00	0.324	0.87	0.251	2.54	0.30 ± 0.11	2.80 ± 2.02
					0.25	0.365	5.00	0.289	5.00	0.286	0.73	0.187	2.16	0.25 ± 0.07	2.63 ± 2.13
					0.125	0.284	5.00	0.502	0.49	0.268	0.59	0.152	1.90	0.31 ± 0.15	0.99 ± 2.10
				31.3	2	0.599	5.00	0.373	5.00	0.478	0.86	0.351	5.00	0.40 ± 0.11	3.62 ± 2.07
					1	0.502	5.00	0.211	5.00	0.355	1.03	0.228	3.98	0.26 ± 0.13	3.34 ± 1.88
					0.5	0.283	5.00	0.072	5.00	0.088	5.00	0.113	3.74	0.09 ± 0.10	4.58 ± 0.63
					0.25	0.123	5.00	0.118	5.00	0.019	5.00	0.085	2.74	0.07 ± 0.05	4.25 ± 1.13
					0.125	0.103	5.00	0.038	5.00	0.079	3.05	0.059	2.28	0.06 ± 0.03	3.44 ± 1.38
AZD1775	Cabozantinib	Wee1	TKI	500	2000	0.847	0.90	0.810	0.60	0.837	0.57	0.978	0.57	0.88 ± 0.07	0.58 ± 0.16
					1000	0.858	0.79	0.769	0.69	0.826	0.54	0.983	0.49	0.86 ± 0.09	0.57 ± 0.14
					500	0.849	0.81	0.742	0.76	0.807	0.56	0.986	0.45	0.85 ± 0.10	0.59 ± 0.17
					250	0.863	0.73	0.782	0.63	0.796	0.57	0.980	0.54	0.85 ± 0.09	0.58 ± 0.09
					125	0.868	0.70	0.725	0.80	0.791	0.57	0.982	0.50	0.83 ± 0.11	0.62 ± 0.13
				250	2000	0.780	0.70	0.551	0.84	0.711	0.61	0.918	0.61	0.73 ± 0.15	0.69 ± 0.11
					1000	0.773	0.64	0.467	1.01	0.644	0.63	0.902	0.66	0.67 ± 0.19	0.77 ± 0.18
					500	0.766	0.63	0.426	1.10	0.585	0.66	0.893	0.69	0.63 ± 0.20	0.82 ± 0.22
					250	0.766	0.61	0.438	1.04	0.601	0.58	0.880	0.73	0.64 ± 0.19	0.79 ± 0.21
					125	0.765	0.60	0.440	1.02	0.567	0.62	0.889	0.70	0.63 ± 0.20	0.78 ± 0.19
				125	2000	0.572	0.83	0.369	0.87	0.461	1.00	0.701	0.78	0.51 ± 0.14	0.88 ± 0.09
					1000	0.505	0.86	0.247	1.18	0.301	1.21	0.599	0.94	0.38 ± 0.17	1.11 ± 0.18
					500	0.485	0.83	0.203	1.31	0.255	1.11	0.551	0.99	0.34 ± 0.17	1.14 ± 0.20
					250	0.479	0.81	0.169	1.49	0.206	1.16	0.548	0.97	0.31 ± 0.19	1.21 ± 0.29
					125	0.444	0.88	0.141	1.71	0.206	1.05	0.544	0.97	0.30 ± 0.19	1.24 ± 0.38
				62.5	2000	0.241	1.43	0.258	0.83	0.241	1.85	0.466	0.88	0.32 ± 0.11	1.19 ± 0.48
					1000	0.169	1.57	0.156	1.07	0.108	2.52	0.315	1.10	0.19 ± 0.09	1.56 ± 0.68
					500	0.125	1.80	0.070	1.83	0.062	2.72	0.211	1.32	0.11 ± 0.07	1.96 ± 0.58
					250	0.130	1.63	0.124	1.04	0.028	3.83	0.189	1.29	0.11 ± 0.07	2.05 ± 1.28
					125	0.136	1.52	0.065	1.70	0.024	3.47	0.179	1.25	0.09 ± 0.07	2.14 ± 1.01
				31.3	2000	0.146	1.50	0.230	0.68	0.153	2.68	0.360	0.86	0.25 ± 0.10	1.40 ± 0.91
					1000	0.048	2.55	0.116	0.90	0.080	2.79	0.165	1.37	0.12 ± 0.05	1.69 ± 0.91
					500	0.039	2.49	0.029	2.21	0.048	2.63	0.103	1.49	0.06 ± 0.03	2.11 ± 0.51
					250	0.042	2.14	-0.002	5.00	0.000	5.00	0.072	1.51	0.02 ± 0.04	3.84 ± 1.85
					125	0.024	3.12	0.003	5.00	0.016	2.93	0.061	1.41	0.03 ± 0.02	3.11 ± 1.47

Agents Tx1	Agents Tx2	Mechanism Tx1	Mechanism Tx2	Conc (ng/ml) Tx1	Conc (ng/ml) Tx2	RD FA	RD CI	SMS-CTR FA	SMS-CTR CI	RH30 FA	RH30 CI	RH41 FA	RH41 CI	Average FA	Average CI
AZD1775	4HC	Wee1	Alkylator	500	5000	0.996	1.07	1.000	0.03	1.000	0.69	1.000	5.00	1.00 ± 0.00	1.91 ± 2.24
					2500	0.934	1.37	1.000	0.03	0.996	0.60	0.996	0.78	1.00 ± 0.03	0.47 ± 0.55
					1250	0.891	1.14	0.830	1.11	0.905	0.78	0.978	0.91	0.90 ± 0.06	0.93 ± 0.17
					625	0.867	0.99	0.815	0.86	0.883	0.59	0.978	0.73	0.89 ± 0.07	0.73 ± 0.17
					313	0.862	0.87	0.833	0.64	0.853	0.55	0.981	0.61	0.89 ± 0.07	0.60 ± 0.14
				250	5000	0.996	1.06	1.000	0.02	1.000	0.63	1.000	0.61	1.00 ± 0.00	0.42 ± 0.43
					2500	0.879	1.46	1.000	0.02	0.973	0.85	0.964	1.12	0.98 ± 0.05	0.66 ± 0.62
					1250	0.823	1.09	0.763	1.15	0.796	0.84	0.866	1.25	0.81 ± 0.04	1.08 ± 0.18
					625	0.811	0.80	0.742	0.81	0.689	0.72	0.845	1.09	0.76 ± 0.07	0.87 ± 0.16
					313	0.798	0.68	0.715	0.65	0.654	0.62	0.847	0.97	0.74 ± 0.09	0.74 ± 0.16
				125	5000	0.993	1.20	1.000	0.04	1.000	0.40	1.000	0.58	1.00 ± 0.00	0.34 ± 0.49
					2500	0.737	1.75	0.989	0.20	0.888	1.09	0.841	1.40	0.91 ± 0.10	0.90 ± 0.66
					1250	0.716	1.08	0.700	1.21	0.534	1.01	0.482	1.72	0.57 ± 0.12	1.31 ± 0.32
					625	0.645	0.85	0.647	0.84	0.361	0.93	0.528	1.29	0.51 ± 0.13	1.02 ± 0.21
					313	0.599	0.74	0.564	0.71	0.267	0.95	0.545	1.10	0.46 ± 0.15	0.92 ± 0.19
				62.5	5000	0.986	1.38	0.990	0.37	1.000	0.61	1.000	5.00	1.00 ± 0.01	1.99 ± 2.15
					2500	0.654	1.77	1.000	0.03	0.639	1.40	0.801	1.26	0.81 ± 0.17	0.90 ± 0.76
					1250	0.602	1.08	0.662	1.24	0.384	1.01	0.279	1.59	0.44 ± 0.18	1.28 ± 0.26
					625	0.423	0.94	0.545	0.94	0.094	1.42	0.334	1.10	0.32 ± 0.19	1.15 ± 0.23
					313	0.287	0.99	0.413	0.81	0.049	1.79	0.283	1.04	0.25 ± 0.15	1.21 ± 0.43
				31.3	5000	0.980	1.51	1.000	0.01	1.000	0.60	1.000	5.00	1.00 ± 0.01	1.87 ± 2.23
					2500	0.623	1.72	0.990	0.18	0.941	0.91	0.734	1.22	0.89 ± 0.17	0.77 ± 0.64
					1250	0.476	1.10	0.601	1.39	0.423	0.85	0.181	1.38	0.40 ± 0.18	1.21 ± 0.26
					625	0.264	0.95	0.505	0.95	0.114	0.88	0.172	1.00	0.26 ± 0.17	0.94 ± 0.05
					313	0.149	1.01	0.380	0.74	0.057	0.95	0.115	0.99	0.18 ± 0.14	0.89 ± 0.12
AZD1775	Bortezomib	Wee1	Proteosome	500	10	0.861	1.07	0.723	1.33	0.721	1.27	0.973	0.88	0.81 ± 0.12	1.16 ± 0.21
					6.67	0.873	0.90	0.753	1.04	0.783	0.90	0.983	0.63	0.84 ± 0.10	0.86 ± 0.17
					4.45	0.872	0.83	0.771	0.87	0.818	0.70	0.988	0.49	0.86 ± 0.09	0.69 ± 0.17
					2.97	0.871	0.78	0.755	0.85	0.826	0.61	0.988	0.47	0.86 ± 0.10	0.64 ± 0.17
					1.98	0.874	0.74	0.786	0.71	0.815	0.59	0.989	0.42	0.86 ± 0.09	0.57 ± 0.14
				250	10	0.783	0.93	0.481	1.65	0.496	1.45	0.882	1.15	0.62 ± 0.20	1.42 ± 0.32
					6.67	0.776	0.82	0.481	1.40	0.530	1.12	0.893	0.95	0.63 ± 0.20	1.16 ± 0.25
					4.45	0.794	0.69	0.530	1.08	0.569	0.89	0.900	0.83	0.67 ± 0.18	0.93 ± 0.16
					2.97	0.798	0.62	0.445	1.23	0.601	0.73	0.912	0.72	0.65 ± 0.21	0.89 ± 0.27
					1.98	0.793	0.60	0.558	0.83	0.549	0.76	0.918	0.66	0.67 ± 0.18	0.75 ± 0.10
				125	10	0.441	1.41	0.459	1.26	0.228	1.88	0.507	1.86	0.40 ± 0.12	1.67 ± 0.31
					6.67	0.484	1.11	0.219	1.89	0.185	1.78	0.517	1.56	0.31 ± 0.17	1.74 ± 0.35
					4.45	0.515	0.92	0.186	1.85	0.210	1.40	0.546	1.30	0.31 ± 0.19	1.52 ± 0.38
					2.97	0.501	0.88	0.192	1.63	0.209	1.25	0.577	1.11	0.33 ± 0.20	1.33 ± 0.32
					1.98	0.506	0.81	0.092	2.75	0.235	1.04	0.595	1.00	0.31 ± 0.23	1.60 ± 0.90
				62.5	10	0.159	2.04	0.246	1.59	0.005	5.00	0.164	2.69	0.14 ± 0.10	3.09 ± 1.52
					6.67	0.158	1.79	0.094	2.29	0.007	5.00	0.148	2.33	0.08 ± 0.07	3.21 ± 1.45
					4.45	0.174	1.50	0.048	3.03	0.003	5.00	0.161	1.91	0.07 ± 0.08	3.31 ± 1.57
					2.97	0.139	1.67	0.038	3.17	0.010	5.00	0.160	1.69	0.07 ± 0.07	3.29 ± 1.58
					1.98	0.153	1.47	0.075	1.79	0.003	5.00	0.179	1.45	0.09 ± 0.08	2.75 ± 1.72
				31.3	10	0.037	3.23	0.204	1.50	0.051	2.41	0.141	2.23	0.13 ± 0.08	2.05 ± 0.71
					6.67	0.051	2.33	0.072	1.97	0.045	1.97	0.050	2.76	0.06 ± 0.01	2.23 ± 0.38
					4.45	0.061	1.86	-0.033	5.00	0.030	1.97	0.037	2.60	0.01 ± 0.04	3.19 ± 1.47
					2.97	0.063	1.68	0.011	4.39	0.008	3.67	0.054	1.84	0.02 ± 0.03	3.30 ± 1.34
					1.98	0.056	1.73	0.142	0.69	0.006	4.22	0.090	1.25	0.08 ± 0.06	2.05 ± 1.56
AZD1775	Etoposide	Wee1	Topo II	500	5000	0.903	1.11	0.931	0.25	0.942	1.57	0.986	0.61	0.95 ± 0.03	0.81 ± 0.58
					1250	0.899	0.70	0.850	0.47	0.895	0.92	0.991	0.39	0.91 ± 0.06	0.59 ± 0.24
					313	0.882	0.68	0.845	0.46	0.827	0.72	0.989	0.41	0.89 ± 0.07	0.53 ± 0.16
					78.3	0.876	0.67	0.781	0.63	0.782	0.66	0.987	0.43	0.85 ± 0.10	0.57 ± 0.12
					19.6	0.876	0.67	0.775	0.65	0.784	0.60	0.987	0.43	0.85 ± 0.10	0.56 ± 0.11
				250	5000	0.874	1.16	0.802	0.54	0.894	2.59	0.969	0.77	0.89 ± 0.07	1.30 ± 0.92
					1250	0.862	0.60	0.714	0.58	0.794	1.47	0.963	0.50	0.82 ± 0.11	0.85 ± 0.46
					313	0.831	0.51	0.713	0.46	0.652	1.02	0.919	0.67	0.76 ± 0.12	0.71 ± 0.25
					78.3	0.817	0.49	0.498	0.91	0.556	0.82	0.893	0.71	0.65 ± 0.19	0.81 ± 0.18
					19.6	0.814	0.48	0.475	0.92	0.529	0.72	0.908	0.63	0.64 ± 0.21	0.76 ± 0.19
				125	5000	0.845	1.32	0.679	1.17	0.864	3.18	0.938	1.21	0.83 ± 0.11	1.85 ± 0.98
					1250	0.792	0.71	0.559	0.99	0.703	2.03	0.849	1.14	0.70 ± 0.13	1.39 ± 0.57
					313	0.755	0.45	0.406	1.09	0.423	1.73	0.573	1.78	0.47 ± 0.16	1.53 ± 0.62
					78.3	0.628	0.56	0.146	3.66	0.200	1.79	0.587	1.07	0.31 ± 0.25	2.17 ± 1.36
					19.6	0.557	0.64	0.083	4.43	0.168	1.38	0.568	0.96	0.27 ± 0.25	2.26 ± 1.75
				62.5	5000	0.810	1.66	0.529	3.40	0.870	3.02	0.888	2.13	0.76 ± 0.17	2.85 ± 0.80
					1250	0.672	1.18	0.489	1.32	0.671	2.19	0.600	3.52	0.59 ± 0.09	2.34 ± 1.08
					313	0.631	0.55	0.226	3.35	0.190	3.96	0.281	4.31	0.23 ± 0.20	3.87 ± 1.71
					78.3	0.300	1.13	-0.023	5.00	0.041	5.00	0.216	2.30	0.08 ± 0.15	4.10 ± 1.96
					19.6	0.234	1.05	-0.018	5.00	0.019	4.99	0.224	1.31	0.08 ± 0.13	3.77 ± 2.21
				31.3	5000	0.779	2.02	0.527	3.36	0.874	2.88	0.877	2.29	0.76 ± 0.16	2.84 ± 0.60
					1250	0.611	1.48	0.351	3.53	0.657	2.27	0.464	5.00	0.49 ± 0.14	3.60 ± 1.54
					313	0.509	0.75	0.119	5.00	0.117	5.00	0.227	5.00	0.15 ± 0.18	5.00 ± 2.12
					78.3	0.205	1.34	0.030	5.00	0.023	5.00	0.126	3.32	0.06 ± 0.09	4.44 ± 1.74
					19.6	0.149	1.02	-0.108	5.00	0.006	5.00	0.071	2.34	-0.01 ± 0.11	4.11 ± 1.99

Agents Tx1	Agents Tx2	Mechanism Tx1	Mechanism Tx2	Conc (ng/ml) Tx1	Conc (ng/ml) Tx2	RD FA	RD CI	SMS-CTR FA	SMS-CTR CI	RH30 FA	RH30 CI	RH41 FA	RH41 CI	Average FA	Average CI
AZD1775	SN-38	*Wee1*	*Topo I*	500	5	**0.916**	*0.55*	**0.920**	*0.29*	**0.935**	*0.37*	**0.990**	*0.38*	**0.95** ± 0.03	*0.35 ± 0.11*
					2.5	**0.893**	*0.63*	**0.910**	*0.30*	**0.880**	*0.51*	**0.987**	*0.44*	**0.93** ± 0.05	*0.42 ± 0.14*
					1.25	**0.871**	*0.72*	**0.886**	*0.36*	**0.858**	*0.50*	**0.987**	*0.43*	**0.91** ± 0.06	*0.43 ± 0.15*
					0.625	**0.867**	*0.72*	**0.867**	*0.40*	**0.837**	*0.51*	**0.989**	*0.39*	**0.90** ± 0.07	*0.43 ± 0.15*
					0.313	**0.862**	*0.73*	**0.839**	*0.48*	**0.827**	*0.50*	**0.986**	*0.44*	**0.88** ± 0.07	*0.47 ± 0.13*
				250	5	**0.869**	*0.48*	**0.841**	*0.30*	**0.836**	*0.68*	**0.931**	*0.62*	**0.87** ± 0.04	*0.53 ± 0.17*
					2.5	**0.838**	*0.51*	**0.792**	*0.35*	**0.783**	*0.61*	**0.912**	*0.66*	**0.83** ± 0.06	*0.54 ± 0.14*
					1.25	**0.820**	*0.52*	**0.757**	*0.38*	**0.720**	*0.59*	**0.903**	*0.67*	**0.79** ± 0.08	*0.55 ± 0.12*
					0.625	**0.807**	*0.52*	**0.754**	*0.37*	**0.663**	*0.59*	**0.903**	*0.66*	**0.77** ± 0.10	*0.54 ± 0.12*
					0.313	**0.800**	*0.52*	**0.633**	*0.57*	**0.626**	*0.59*	**0.908**	*0.63*	**0.72** ± 0.14	*0.59 ± 0.04*
				125	5	**0.799**	*0.52*	**0.718**	*0.36*	**0.773**	*0.83*	**0.693**	*1.05*	**0.73** ± 0.05	*0.75 ± 0.31*
					2.5	**0.778**	*0.44*	**0.637**	*0.39*	**0.695**	*0.71*	**0.571**	*1.18*	**0.63** ± 0.09	*0.76 ± 0.36*
					1.25	**0.741**	*0.43*	**0.629**	*0.34*	**0.561**	*0.75*	**0.537**	*1.12*	**0.58** ± 0.09	*0.74 ± 0.35*
					0.625	**0.727**	*0.40*	**0.615**	*0.33*	**0.514**	*0.62*	**0.583**	*0.94*	**0.57** ± 0.09	*0.63 ± 0.28*
					0.313	**0.636**	*0.52*	**0.450**	*0.52*	**0.369**	*0.77*	**0.588**	*0.90*	**0.47** ± 0.12	*0.73 ± 0.19*
				62.5	5	**0.697**	*0.72*	**0.580**	*0.49*	**0.776**	*0.74*	**0.407**	*1.57*	**0.59** ± 0.16	*0.93 ± 0.48*
					2.5	**0.628**	*0.64*	**0.563**	*0.35*	**0.674**	*0.66*	**0.304**	*1.48*	**0.51** ± 0.17	*0.83 ± 0.49*
					1.25	**0.646**	*0.41*	**0.526**	*0.30*	**0.521**	*0.70*	**0.321**	*1.09*	**0.46** ± 0.13	*0.69 ± 0.35*
					0.625	**0.576**	*0.42*	**0.512**	*0.26*	**0.310**	*0.96*	**0.323**	*0.93*	**0.38** ± 0.13	*0.71 ± 0.36*
					0.313	**0.337**	*0.83*	**0.246**	*0.60*	**0.107**	*2.01*	**0.257**	*1.02*	**0.20** ± 0.10	*1.21 ± 0.62*
				31.3	5	**0.598**	*1.06*	**0.485**	*0.61*	**0.789**	*0.65*	**0.363**	*1.44*	**0.55** ± 0.18	*0.90 ± 0.39*
					2.5	**0.564**	*0.70*	**0.474**	*0.38*	**0.723**	*0.49*	**0.277**	*1.17*	**0.49** ± 0.19	*0.68 ± 0.35*
					1.25	**0.540**	*0.47*	**0.431**	*0.29*	**0.535**	*0.58*	**0.202**	*1.05*	**0.39** ± 0.16	*0.64 ± 0.32*
					0.625	**0.513**	*0.35*	**0.437**	*0.21*	**0.261**	*0.99*	**0.189**	*0.84*	**0.30** ± 0.15	*0.68 ± 0.38*
					0.313	**0.221**	*0.95*	**0.151**	*0.61*	**0.039**	*4.41*	**0.130**	*0.91*	**0.11** ± 0.08	*1.98 ± 1.80*
Bortezomib	4HC	*Proteosome*	*Alkylator*	10	5000	**0.968**	*1.90*	**1.000**	*0.03*	**1.000**	*0.77*	**0.999**	*0.84*	**1.00** ± 0.02	*0.54 ± 0.77*
					2500	**0.402**	*2.52*	**1.000**	*0.05*	**0.876**	*1.43*	**0.699**	*1.73*	**0.86** ± 0.26	*1.07 ± 1.03*
					1250	**0.358**	*1.61*	**0.554**	*2.18*	**0.091**	*2.41*	**0.073**	*2.91*	**0.24** ± 0.23	*2.50 ± 0.54*
					625	**0.209**	*1.31*	**0.454**	*1.76*	**0.026**	*2.69*	**0.053**	*2.71*	**0.18** ± 0.20	*2.39 ± 0.70*
					313	**0.131**	*1.15*	**0.333**	*1.62*	**0.014**	*2.81*	**0.063**	*2.32*	**0.14** ± 0.14	*2.25 ± 0.74*
				6.67	5000	**0.959**	*1.93*	**1.000**	*0.03*	**0.793**	*2.58*	**1.000**	*0.68*	**0.93** ± 0.10	*1.09 ± 1.16*
					2500	**0.361**	*2.42*	**1.000**	*0.06*	**0.798**	*1.44*	**0.541**	*1.75*	**0.78** ± 0.28	*1.08 ± 0.99*
					1250	**0.342**	*1.43*	**0.521**	*2.10*	**0.139**	*1.75*	**0.013**	*3.66*	**0.22** ± 0.22	*2.50 ± 0.99*
					625	**0.178**	*1.12*	**0.349**	*1.89*	**0.018**	*2.19*	**0.004**	*4.51*	**0.12** ± 0.16	*2.86 ± 1.46*
					313	**0.101**	*0.94*	**0.232**	*1.68*	**0.005**	*2.71*	**-0.002**	*5.00*	**0.08** ± 0.11	*3.13 ± 1.77*
				4.45	5000	**0.958**	*1.88*	**1.000**	*0.03*	**1.000**	*5.00*	**1.000**	*0.75*	**1.00** ± 0.02	*1.93 ± 2.19*
					2500	**0.375**	*2.26*	**1.000**	*0.02*	**0.673**	*1.51*	**0.576**	*1.53*	**0.75** ± 0.26	*1.02 ± 0.94*
					1250	**0.318**	*1.33*	**0.464**	*2.22*	**0.142**	*1.47*	**0.021**	*2.52*	**0.21** ± 0.19	*2.07 ± 0.58*
					625	**0.207**	*0.92*	**0.316**	*1.84*	**0.037**	*1.42*	**0.023**	*1.89*	**0.13** ± 0.14	*1.72 ± 0.45*
					313	**0.066**	*0.83*	**0.191**	*1.63*	**0.036**	*1.13*	**-0.002**	*5.00*	**0.07** ± 0.08	*2.59 ± 1.93*
				2.97	5000	**0.930**	*2.08*	**0.976**	*0.68*	**1.000**	*0.47*	**1.000**	*0.70*	**0.99** ± 0.03	*0.62 ± 0.74*
					2500	**0.406**	*2.11*	**1.000**	*0.03*	**0.776**	*1.30*	**0.474**	*1.53*	**0.75** ± 0.28	*0.95 ± 0.88*
					1250	**0.319**	*1.24*	**0.449**	*2.19*	**0.189**	*1.21*	**0.010**	*2.49*	**0.22** ± 0.19	*1.96 ± 0.66*
					625	**0.148**	*0.89*	**0.332**	*1.62*	**0.055**	*1.04*	**0.016**	*1.61*	**0.13** ± 0.14	*1.42 ± 0.38*
					313	**0.056**	*0.72*	**0.228**	*1.28*	**0.059**	*0.75*	**-0.018**	*5.00*	**0.09** ± 0.10	*2.34 ± 2.06*
				1.98	5000	**0.956**	*1.84*	**1.000**	*0.04*	**1.000**	*5.00*	**1.000**	*0.71*	**1.00** ± 0.02	*1.92 ± 2.20*
					2500	**0.399**	*2.07*	**1.000**	*0.02*	**0.823**	*1.19*	**0.352**	*1.58*	**0.73** ± 0.32	*0.93 ± 0.88*
					1250	**0.315**	*1.18*	**0.463**	*2.04*	**0.201**	*1.09*	**0.032**	*1.60*	**0.23** ± 0.18	*1.58 ± 0.44*
					625	**0.144**	*0.82*	**0.340**	*1.50*	**0.120**	*0.74*	**0.011**	*1.39*	**0.16** ± 0.14	*1.21 ± 0.39*
					313	**0.054**	*0.62*	**0.219**	*1.20*	**0.100**	*0.52*	**-0.012**	*5.00*	**0.10** ± 0.10	*2.24 ± 2.13*
Bortezomib	Etoposide	*Proteosome*	*Topo II*	10	5000	**0.569**	*5.00*	**0.571**	*2.98*	**0.871**	*3.35*	**0.769**	*5.00*	**0.74** ± 0.15	*3.78 ± 1.07*
					1250	**0.375**	*5.00*	**0.540**	*1.44*	**0.584**	*3.54*	**0.313**	*5.00*	**0.48** ± 0.13	*3.33 ± 1.68*
					313	**0.264**	*2.86*	**0.338**	*1.88*	**-0.016**	*5.00*	**0.167**	*5.00*	**0.16** ± 0.15	*3.96 ± 1.57*
					78.3	**0.001**	*5.00*	**0.335**	*1.19*	**-0.027**	*5.00*	**0.090**	*5.00*	**0.13** ± 0.16	*3.73 ± 1.91*
					19.6	**-0.032**	*5.00*	**0.225**	*1.32*	**-0.017**	*5.00*	**0.063**	*3.60*	**0.09** ± 0.12	*3.31 ± 1.74*
				6.67	5000	**0.570**	*5.00*	**0.486**	*5.00*	**0.892**	*2.73*	**0.805**	*4.38*	**0.73** ± 0.19	*4.04 ± 1.07*
					1250	**0.387**	*4.68*	**0.498**	*1.52*	**0.615**	*3.00*	**0.370**	*5.00*	**0.49** ± 0.11	*3.17 ± 1.61*
					313	**0.253**	*2.82*	**0.374**	*1.29*	**0.002**	*5.00*	**0.115**	*5.00*	**0.16** ± 0.16	*3.76 ± 1.81*
					78.3	**-0.068**	*5.00*	**0.092**	*5.00*	**-0.040**	*5.00*	**0.040**	*5.00*	**0.03** ± 0.07	*5.00 ± 0.00*
					19.6	**-0.074**	*5.00*	**0.006**	*5.00*	**-0.014**	*5.00*	**-0.038**	*5.00*	**-0.02** ± 0.03	*5.00 ± 0.00*
				4.45	5000	**0.578**	*5.00*	**0.483**	*4.98*	**0.889**	*2.70*	**0.845**	*3.19*	**0.74** ± 0.20	*3.62 ± 1.20*
					1250	**0.378**	*4.77*	**0.367**	*3.33*	**0.634**	*2.68*	**0.426**	*5.00*	**0.48** ± 0.12	*3.67 ± 1.12*
					313	**0.230**	*3.08*	**0.265**	*2.33*	**0.012**	*5.00*	**0.141**	*5.00*	**0.14** ± 0.11	*4.11 ± 1.36*
					78.3	**-0.042**	*5.00*	**0.011**	*5.00*	**-0.029**	*5.00*	**0.020**	*5.00*	**0.00** ± 0.03	*5.00 ± 0.00*
					19.6	**-0.070**	*5.00*	**-0.027**	*5.00*	**0.018**	*3.03*	**-0.024**	*5.00*	**-0.01** ± 0.04	*4.34 ± 0.99*
				2.97	5000	**0.582**	*5.00*	**0.492**	*4.54*	**0.893**	*2.55*	**0.858**	*2.78*	**0.75** ± 0.20	*3.29 ± 1.23*
					1250	**0.391**	*4.38*	**0.414**	*2.25*	**0.620**	*2.73*	**0.447**	*5.00*	**0.49** ± 0.10	*3.33 ± 1.31*
					313	**0.224**	*3.12*	**0.152**	*5.00*	**-0.012**	*5.00*	**0.166**	*5.00*	**0.10** ± 0.10	*5.00 ± 0.94*
					78.3	**-0.047**	*5.00*	**0.199**	*1.33*	**-0.007**	*5.00*	**-0.001**	*5.00*	**0.06** ± 0.11	*3.78 ± 1.84*
					19.6	**-0.084**	*5.00*	**0.116**	*1.30*	**-0.014**	*5.00*	**-0.029**	*5.00*	**0.02** ± 0.08	*3.77 ± 1.85*
				1.98	5000	**0.589**	*5.00*	**0.517**	*3.69*	**0.894**	*2.49*	**0.859**	*2.72*	**0.76** ± 0.19	*2.97 ± 1.14*
					1250	**0.402**	*4.09*	**0.380**	*2.81*	**0.624**	*2.63*	**0.450**	*5.00*	**0.48** ± 0.11	*3.48 ± 1.12*
					313	**0.249**	*2.57*	**0.199**	*4.10*	**0.005**	*5.00*	**0.146**	*5.00*	**0.12** ± 0.11	*4.70 ± 1.15*
					78.3	**-0.018**	*5.00*	**0.111**	*4.04*	**-0.013**	*5.00*	**0.004**	*5.00*	**0.03** ± 0.06	*4.68 ± 0.48*
					19.6	**-0.054**	*5.00*	**0.075**	*2.51*	**0.010**	*4.01*	**-0.045**	*5.00*	**0.01** ± 0.06	*3.84 ± 1.18*

Agents Tx1	Agents Tx2	Mechanism Tx1	Mechanism Tx2	Conc (ng/ml) Tx1	Conc (ng/ml) Tx2	RD FA	RD CI	SMS-CTR FA	SMS-CTR CI	RH30 FA	RH30 CI	RH41 FA	RH41 CI	Average FA	Average CI
AZD1775	Vinorelbine	*Wee1*	*Microtubule*	500	4	**0.911**	*1.18*	**0.920**	*1.04*	**0.829**	*0.47*	**0.994**	*0.30*	**0.91** ± 0.07	*0.61 ± 0.43*
					2	**0.894**	*0.99*	**0.883**	*0.93*	**0.815**	*0.51*	**0.992**	*0.35*	**0.90** ± 0.07	*0.60 ± 0.31*
					1	**0.880**	*0.88*	**0.867**	*0.73*	**0.795**	*0.55*	**0.991**	*0.36*	**0.88** ± 0.08	*0.55 ± 0.22*
					0.5	**0.862**	*0.86*	**0.858**	*0.60*	**0.790**	*0.56*	**0.988**	*0.42*	**0.88** ± 0.08	*0.53 ± 0.19*
					0.25	**0.852**	*0.85*	**0.833**	*0.59*	**0.776**	*0.60*	**0.988**	*0.41*	**0.87** ± 0.09	*0.53 ± 0.18*
				250	4	**0.890**	*1.15*	**0.880**	*1.37*	**0.802**	*0.28*	**0.988**	*0.26*	**0.89** ± 0.08	*0.63 ± 0.58*
					2	**0.869**	*0.87*	**0.880**	*0.78*	**0.786**	*0.29*	**0.988**	*0.23*	**0.88** ± 0.08	*0.43 ± 0.33*
					1	**0.841**	*0.73*	**0.831**	*0.68*	**0.747**	*0.34*	**0.984**	*0.26*	**0.85** ± 0.10	*0.42 ± 0.24*
					0.5	**0.832**	*0.61*	**0.790**	*0.58*	**0.734**	*0.35*	**0.978**	*0.30*	**0.83** ± 0.10	*0.41 ± 0.16*
					0.25	**0.827**	*0.53*	**0.793**	*0.44*	**0.738**	*0.35*	**0.980**	*0.27*	**0.84** ± 0.10	*0.35 ± 0.11*
				125	4	**0.847**	*1.44*	**0.896**	*1.10*	**0.582**	*0.36*	**0.934**	*0.57*	**0.80** ± 0.16	*0.68 ± 0.49*
					2	**0.688**	*1.95*	**0.791**	*1.25*	**0.367**	*0.76*	**0.802**	*1.03*	**0.65** ± 0.20	*1.01 ± 0.51*
					1	**0.526**	*2.20*	**0.469**	*2.62*	**0.203**	*1.57*	**0.611**	*1.51*	**0.43** ± 0.18	*1.90 ± 0.53*
					0.5	**0.494**	*1.60*	**0.323**	*2.65*	**0.184**	*1.45*	**0.553**	*1.37*	**0.35** ± 0.17	*1.82 ± 0.60*
					0.25	**0.513**	*1.10*	**0.151**	*4.00*	**0.181**	*1.27*	**0.548**	*1.16*	**0.29** ± 0.21	*2.14 ± 1.41*
				62.5	4	**0.740**	*2.56*	**0.874**	*1.30*	**0.521**	*0.30*	**0.906**	*0.62*	**0.77** ± 0.17	*0.74 ± 1.00*
					2	**0.473**	*4.16*	**0.714**	*1.74*	**0.320**	*0.66*	**0.632**	*1.64*	**0.56** ± 0.17	*1.35 ± 1.49*
					1	**0.361**	*3.54*	**0.445**	*2.61*	**0.205**	*1.08*	**0.451**	*1.87*	**0.37** ± 0.11	*1.85 ± 1.05*
					0.5	**0.281**	*2.88*	**0.170**	*4.94*	**0.191**	*0.86*	**0.428**	*1.32*	**0.26** ± 0.12	*2.37 ± 1.84*
					0.25	**0.286**	*1.77*	**0.192**	*2.48*	**0.194**	*0.67*	**0.446**	*0.92*	**0.28** ± 0.12	*1.36 ± 0.83*
				31.3	4	**0.809**	*1.68*	**0.817**	*1.93*	**0.447**	*0.34*	**0.838**	*0.95*	**0.70** ± 0.19	*1.07 ± 0.72*
					2	**0.449**	*4.36*	**0.630**	*2.40*	**0.163**	*2.44*	**0.320**	*4.85*	**0.37** ± 0.20	*3.23 ± 1.28*
					1	**0.207**	*5.00*	**0.155**	*5.00*	**0.034**	*5.00*	**0.120**	*5.00*	**0.10** ± 0.07	*5.00 ± 0.00*
					0.5	**0.133**	*5.00*	**-0.057**	*5.00*	**0.010**	*5.00*	**0.102**	*5.00*	**0.02** ± 0.09	*5.00 ± 0.00*
					0.25	**0.130**	*3.56*	**-0.058**	*5.00*	**0.001**	*5.00*	**0.084**	*3.72*	**0.01** ± 0.08	*4.57 ± 0.79*
Bortezomib	Actinomycin D	*Proteosome*	*Alkylator*	10	2	**0.562**	*5.00*	**0.398**	*5.00*	**0.187**	*5.00*	**0.300**	*5.00*	**0.30** ± 0.16	*5.00 ± 0.00*
					1	**0.466**	*5.00*	**0.271**	*5.00*	**0.126**	*5.00*	**0.224**	*4.82*	**0.21** ± 0.14	*4.94 ± 0.09*
					0.5	**0.232**	*5.00*	**0.175**	*5.00*	**-0.039**	*5.00*	**0.086**	*5.00*	**0.07** ± 0.12	*5.00 ± 0.00*
					0.25	**0.058**	*5.00*	**0.167**	*5.00*	**-0.058**	*5.00*	**0.061**	*4.40*	**0.06** ± 0.09	*4.80 ± 0.30*
					0.125	**0.029**	*5.00*	**0.233**	*5.00*	**-0.046**	*5.00*	**0.050**	*3.55*	**0.08** ± 0.12	*4.52 ± 0.72*
				6.67	2	**0.585**	*5.00*	**0.353**	*5.00*	**0.171**	*5.00*	**0.273**	*5.00*	**0.27** ± 0.18	*5.00 ± 0.00*
					1	**0.485**	*5.00*	**0.255**	*5.00*	**0.094**	*5.00*	**0.137**	*5.00*	**0.16** ± 0.18	*5.00 ± 0.00*
					0.5	**0.230**	*5.00*	**0.053**	*5.00*	**-0.051**	*5.00*	**-0.024**	*5.00*	**-0.01** ± 0.13	*5.00 ± 0.00*
					0.25	**0.031**	*5.00*	**0.076**	*5.00*	**-0.050**	*5.00*	**-0.026**	*5.00*	**0.00** ± 0.06	*5.00 ± 0.00*
					0.125	**0.020**	*5.00*	**0.023**	*5.00*	**-0.029**	*5.00*	**-0.028**	*5.00*	**-0.01** ± 0.03	*5.00 ± 0.00*
				4.45	2	**0.583**	*5.00*	**0.394**	*5.00*	**0.194**	*5.00*	**0.277**	*5.00*	**0.29** ± 0.17	*5.00 ± 0.00*
					1	**0.466**	*5.00*	**0.246**	*5.00*	**0.115**	*5.00*	**0.152**	*5.00*	**0.17** ± 0.16	*5.00 ± 0.00*
					0.5	**0.240**	*5.00*	**0.069**	*5.00*	**-0.054**	*5.00*	**0.011**	*5.00*	**0.01** ± 0.13	*5.00 ± 0.00*
					0.25	**0.065**	*5.00*	**0.125**	*5.00*	**-0.052**	*5.00*	**-0.038**	*5.00*	**0.01** ± 0.08	*5.00 ± 0.00*
					0.125	**0.040**	*5.00*	**0.042**	*5.00*	**-0.015**	*5.00*	**-0.055**	*5.00*	**-0.01** ± 0.05	*5.00 ± 0.00*
				2.97	2	**0.603**	*5.00*	**0.425**	*5.00*	**0.202**	*5.00*	**0.269**	*5.00*	**0.30** ± 0.18	*5.00 ± 0.00*
					1	**0.476**	*5.00*	**0.322**	*5.00*	**0.093**	*5.00*	**0.136**	*5.00*	**0.18** ± 0.18	*5.00 ± 0.00*
					0.5	**0.266**	*5.00*	**0.128**	*5.00*	**-0.049**	*5.00*	**0.006**	*5.00*	**0.03** ± 0.14	*5.00 ± 0.00*
					0.25	**0.080**	*5.00*	**0.044**	*5.00*	**-0.039**	*5.00*	**-0.012**	*5.00*	**0.00** ± 0.05	*5.00 ± 0.00*
					0.125	**0.052**	*5.00*	**0.144**	*5.00*	**-0.013**	*5.00*	**-0.036**	*5.00*	**0.03** ± 0.08	*5.00 ± 0.00*
				1.98	2	**0.591**	*5.00*	**0.464**	*5.00*	**0.221**	*5.00*	**0.264**	*5.00*	**0.32** ± 0.17	*5.00 ± 0.00*
					1	**0.478**	*5.00*	**0.467**	*3.35*	**0.141**	*5.00*	**0.157**	*4.97*	**0.25** ± 0.19	*4.44 ± 0.82*
					0.5	**0.278**	*5.00*	**0.133**	*5.00*	**-0.036**	*5.00*	**0.026**	*5.00*	**0.04** ± 0.08	*5.00 ± 0.00*
					0.25	**0.094**	*5.00*	**0.114**	*5.00*	**-0.036**	*5.00*	**-0.026**	*5.00*	**0.02** ± 0.08	*5.00 ± 0.00*
					0.125	**0.050**	*5.00*	**0.045**	*5.00*	**0.006**	*5.00*	**-0.006**	*5.00*	**0.01** ± 0.03	*5.00 ± 0.00*
Bortezomib	SN-38	*Proteosome*	*Topo I*	10	5	**0.509**	*1.98*	**0.510**	*1.18*	**0.792**	*1.09*	**0.342**	*2.21*	**0.55** ± 0.19	*1.49 ± 0.56*
					2.5	**0.404**	*1.84*	**0.557**	*0.86*	**0.698**	*1.05*	**0.173**	*2.62*	**0.48** ± 0.22	*1.51 ± 0.80*
					1.25	**0.414**	*1.16*	**0.545**	*0.79*	**0.521**	*1.22*	**0.112**	*2.57*	**0.39** ± 0.20	*1.53 ± 0.78*
					0.625	**0.358**	*1.00*	**0.444**	*0.88*	**0.138**	*2.99*	**0.087**	*2.41*	**0.22** ± 0.17	*2.09 ± 1.04*
					0.313	**0.304**	*0.91*	**0.312**	*1.05*	**-0.069**	*5.00*	**0.076**	*2.27*	**0.11** ± 0.19	*2.77 ± 1.90*
				6.67	5	**0.505**	*1.84*	**0.499**	*0.97*	**0.806**	*0.87*	**0.360**	*1.77*	**0.56** ± 0.19	*1.20 ± 0.52*
					2.5	**0.461**	*1.30*	**0.378**	*1.00*	**0.706**	*0.85*	**0.161**	*2.26*	**0.41** ± 0.23	*1.37 ± 0.63*
					1.25	**0.405**	*1.01*	**0.344**	*0.87*	**0.543**	*0.93*	**0.049**	*3.31*	**0.31** ± 0.21	*1.70 ± 1.19*
					0.625	**0.349**	*0.83*	**0.442**	*0.61*	**0.170**	*2.14*	**0.014**	*4.90*	**0.21** ± 0.19	*2.55 ± 1.97*
					0.313	**0.300**	*0.71*	**0.423**	*0.59*	**-0.060**	*5.00*	**-0.003**	*5.00*	**0.12** ± 0.23	*3.53 ± 2.51*
				4.45	5	**0.484**	*1.90*	**0.416**	*1.06*	**0.807**	*0.76*	**0.436**	*1.26*	**0.55** ± 0.18	*1.03 ± 0.48*
					2.5	**0.450**	*1.23*	**0.355**	*0.87*	**0.707**	*0.72*	**0.205**	*1.60*	**0.42** ± 0.21	*1.06 ± 0.39*
					1.25	**0.408**	*0.87*	**0.310**	*0.73*	**0.545**	*0.78*	**0.077**	*2.09*	**0.31** ± 0.20	*1.20 ± 0.65*
					0.625	**0.394**	*0.59*	**0.260**	*0.67*	**0.134**	*2.37*	**0.058**	*1.74*	**0.15** ± 0.15	*1.59 ± 0.86*
					0.313	**0.261**	*0.66*	**0.139**	*0.87*	**-0.069**	*5.00*	**0.011**	*3.20*	**0.03** ± 0.15	*3.02 ± 2.06*
				2.97	5	**0.549**	*1.34*	**0.410**	*0.96*	**0.805**	*0.70*	**0.492**	*0.97*	**0.57** ± 0.17	*0.88 ± 0.26*
					2.5	**0.429**	*1.27*	**0.357**	*0.73*	**0.703**	*0.65*	**0.233**	*1.26*	**0.43** ± 0.20	*0.88 ± 0.33*
					1.25	**0.397**	*0.83*	**0.333**	*0.54*	**0.530**	*0.71*	**0.093**	*1.58*	**0.32** ± 0.18	*0.94 ± 0.46*
					0.625	**0.390**	*0.51*	**0.260**	*0.51*	**0.132**	*2.22*	**0.044**	*1.70*	**0.15** ± 0.15	*1.48 ± 0.86*
					0.313	**0.304**	*0.47*	**0.148**	*0.63*	**-0.057**	*5.00*	**0.033**	*1.40*	**0.04** ± 0.16	*2.34 ± 2.12*
				1.98	5	**0.519**	*1.49*	**0.390**	*0.96*	**0.798**	*0.68*	**0.505**	*0.85*	**0.56** ± 0.17	*0.83 ± 0.35*
					2.5	**0.421**	*1.27*	**0.362**	*0.63*	**0.702**	*0.60*	**0.231**	*1.15*	**0.43** ± 0.20	*0.79 ± 0.35*
					1.25	**0.396**	*0.77*	**0.303**	*0.50*	**0.521**	*0.66*	**0.089**	*1.46*	**0.30** ± 0.18	*0.88 ± 0.42*
					0.625	**0.389**	*0.46*	**0.247**	*0.43*	**0.131**	*2.13*	**0.053**	*1.29*	**0.14** ± 0.15	*1.28 ± 0.81*
					0.313	**0.282**	*0.45*	**0.217**	*0.36*	**-0.003**	*5.00*	**0.006**	*3.40*	**0.07** ± 0.15	*2.92 ± 2.29*

Agents		Mechanism		Conc (ng/ml)		RD		SMS-CTR		RH30		RH41		Average	
Tx1	Tx2	Tx1	Tx2	Tx1	Tx2	FA	CI	FA	CI	FA	CI	FA	CI	FA	CI
Bortezomib	Vinorelbine	Proteosome	Microtubule	10	4	0.764	2.51	0.853	1.88	0.435	1.02	0.667	2.81	0.65 ± 0.18	1.90 ± 0.79
					2	0.325	5.00	0.636	2.88	0.029	5.00	0.223	5.00	0.30 ± 0.25	4.29 ± 1.06
					1	0.087	5.00	0.381	3.90	-0.015	5.00	0.084	5.00	0.15 ± 0.17	4.63 ± 0.55
					0.5	0.057	5.00	0.207	4.57	-0.015	5.00	0.094	5.00	0.10 ± 0.09	4.86 ± 0.22
					0.25	0.035	5.00	0.223	2.70	-0.024	5.00	0.056	5.00	0.09 ± 0.11	4.23 ± 1.15
				6.67	4	0.732	2.77	0.856	1.71	0.463	0.70	0.661	2.65	0.66 ± 0.16	1.69 ± 0.96
					2	0.259	5.00	0.575	3.34	0.035	5.00	0.122	5.00	0.24 ± 0.24	4.45 ± 0.83
					1	0.062	5.00	0.173	5.00	-0.003	5.00	-0.008	5.00	0.05 ± 0.08	5.00 ± 0.00
					0.5	-0.037	5.00	0.046	5.00	-0.010	5.00	-0.017	5.00	0.01 ± 0.04	5.00 ± 0.00
					0.25	-0.045	5.00	0.157	3.21	-0.021	5.00	-0.028	5.00	0.04 ± 0.09	4.40 ± 0.90
				4.45	4	0.735	2.64	0.854	1.65	0.449	0.56	0.659	2.52	0.65 ± 0.17	1.58 ± 0.96
					2	0.291	5.00	0.592	3.00	0.055	5.00	0.149	5.00	0.27 ± 0.23	4.33 ± 1.00
					1	-0.008	5.00	0.170	5.00	-0.005	5.00	0.014	5.00	0.06 ± 0.09	5.00 ± 0.00
					0.5	-0.040	5.00	0.092	5.00	-0.008	5.00	-0.015	5.00	0.02 ± 0.06	5.00 ± 0.00
					0.25	-0.051	5.00	0.097	4.60	-0.017	5.00	-0.037	5.00	0.01 ± 0.07	4.87 ± 0.20
				2.97	4	0.731	2.64	0.860	1.52	0.444	0.46	0.658	2.44	0.65 ± 0.17	1.47 ± 1.00
					2	0.272	5.00	0.587	2.96	0.047	5.00	0.086	5.00	0.24 ± 0.25	4.32 ± 1.02
					1	0.010	5.00	0.173	5.00	0.003	5.00	-0.007	5.00	0.06 ± 0.09	5.00 ± 0.00
					0.5	-0.058	5.00	0.023	5.00	-0.001	5.00	-0.006	5.00	0.01 ± 0.03	5.00 ± 0.00
					0.25	-0.059	5.00	0.132	3.23	-0.012	5.00	-0.032	5.00	0.03 ± 0.09	4.41 ± 0.89
				1.98	4	0.745	2.42	0.856	1.53	0.449	0.38	0.670	2.25	0.66 ± 0.17	1.39 ± 0.93
					2	0.286	5.00	0.604	2.71	0.092	5.00	0.151	5.00	0.28 ± 0.23	4.24 ± 1.15
					1	0.026	5.00	0.174	5.00	-0.009	5.00	0.024	5.00	0.06 ± 0.08	5.00 ± 0.00
					0.5	-0.028	5.00	0.053	5.00	0.025	5.00	-0.022	5.00	0.02 ± 0.04	5.00 ± 0.00
					0.25	-0.072	5.00	0.014	5.00	-0.023	5.00	-0.036	5.00	-0.01 ± 0.04	5.00 ± 0.00
Cabozantinib	Actinomycin D	TKI	Alkylator	2000	2	0.615	5.00	0.393	5.00	0.249	5.00	0.396	4.64	0.35 ± 0.15	4.88 ± 0.18
					1	0.553	5.00	0.249	5.00	0.220	4.35	0.346	2.94	0.27 ± 0.15	4.10 ± 0.97
					0.5	0.354	5.00	0.192	5.00	0.151	5.00	0.308	1.99	0.22 ± 0.10	4.00 ± 1.51
					0.25	0.130	5.00	0.224	5.00	0.046	5.00	0.283	1.45	0.18 ± 0.10	3.82 ± 1.78
					0.125	0.073	5.00	0.313	2.30	0.104	5.00	0.328	0.90	0.25 ± 0.13	2.73 ± 2.05
				1000	2	0.601	5.00	0.354	5.00	0.199	5.00	0.324	5.00	0.29 ± 0.17	5.00 ± 0.00
					1	0.521	5.00	0.235	5.00	0.163	5.00	0.212	4.24	0.20 ± 0.16	4.75 ± 0.38
					0.5	0.299	5.00	0.084	5.00	-0.019	5.00	0.144	3.38	0.07 ± 0.13	4.46 ± 0.81
					0.25	0.096	5.00	0.224	5.00	-0.035	5.00	0.085	3.47	0.09 ± 0.11	4.49 ± 0.77
					0.125	0.062	5.00	0.008	5.00	0.005	5.00	0.105	2.08	0.04 ± 0.05	4.03 ± 1.46
				500	2	0.607	5.00	0.373	5.00	0.196	5.00	0.277	5.00	0.28 ± 0.18	5.00 ± 0.00
					1	0.499	5.00	0.220	5.00	0.146	5.00	0.173	4.69	0.18 ± 0.16	4.90 ± 0.16
					0.5	0.309	5.00	0.071	5.00	-0.028	5.00	0.064	5.00	0.04 ± 0.14	5.00 ± 0.00
					0.25	0.048	5.00	0.012	5.00	-0.050	5.00	0.004	5.00	-0.01 ± 0.04	5.00 ± 0.00
					0.125	0.011	5.00	-0.026	5.00	-0.014	5.00	0.018	5.00	-0.01 ± 0.02	5.00 ± 0.00
				250	2	0.583	5.00	0.362	5.00	0.180	5.00	0.271	5.00	0.27 ± 0.17	5.00 ± 0.00
					1	0.497	5.00	0.306	5.00	0.120	5.00	0.165	4.68	0.20 ± 0.17	4.89 ± 0.16
					0.5	0.294	5.00	0.054	5.00	-0.023	5.00	0.032	5.00	0.02 ± 0.14	5.00 ± 0.00
					0.25	0.119	5.00	0.155	5.00	-0.062	5.00	-0.026	5.00	0.02 ± 0.11	5.00 ± 0.00
					0.125	0.010	5.00	0.051	5.00	-0.018	5.00	0.006	5.00	0.01 ± 0.03	5.00 ± 0.00
				125	2	0.607	5.00	0.379	5.00	0.187	5.00	0.284	5.00	0.28 ± 0.18	5.00 ± 0.00
					1	0.485	5.00	0.304	5.00	0.164	5.00	0.165	4.58	0.21 ± 0.15	4.86 ± 0.21
					0.5	0.295	5.00	0.058	5.00	-0.007	5.00	0.013	5.00	0.02 ± 0.14	5.00 ± 0.00
					0.25	0.113	5.00	0.022	5.00	-0.065	5.00	-0.019	5.00	-0.02 ± 0.08	5.00 ± 0.00
					0.125	0.033	5.00	-0.005	5.00	-0.019	5.00	-0.007	5.00	-0.01 ± 0.02	5.00 ± 0.00
Cabozantinib	Etoposide	TKI	Topo II	2000	5000	0.677	3.95	0.486	4.69	0.924	1.80	0.851	2.86	0.75 ± 0.20	3.12 ± 1.26
					1250	0.439	3.62	0.347	3.76	0.735	1.80	0.529	4.50	0.54 ± 0.17	3.35 ± 1.15
					313	0.356	1.69	0.222	3.39	0.278	3.46	0.317	3.46	0.27 ± 0.06	3.44 ± 0.87
					78.3	0.109	3.13	0.153	2.43	0.128	4.18	0.300	1.44	0.19 ± 0.09	2.68 ± 1.15
					19.6	0.078	2.09	0.189	0.78	0.143	2.84	0.269	1.01	0.20 ± 0.08	1.54 ± 0.96
				1000	5000	0.641	4.73	0.455	5.00	0.923	1.80	0.847	2.95	0.74 ± 0.21	3.25 ± 1.52
					1250	0.462	3.08	0.362	3.19	0.726	1.77	0.471	5.00	0.52 ± 0.16	3.32 ± 1.33
					313	0.337	1.63	0.169	5.00	0.168	5.00	0.215	5.00	0.18 ± 0.08	5.00 ± 1.69
					78.3	0.029	5.00	0.053	5.00	0.031	5.00	0.142	3.12	0.08 ± 0.05	4.37 ± 0.94
					19.6	0.036	3.39	0.050	5.00	0.056	3.96	0.119	1.81	0.07 ± 0.04	3.59 ± 1.33
				500	5000	0.659	4.22	0.450	5.00	0.910	2.09	0.845	2.98	0.74 ± 0.21	3.36 ± 1.29
					1250	0.453	3.13	0.315	4.72	0.677	2.11	0.417	5.00	0.47 ± 0.15	3.94 ± 1.36
					313	0.315	1.72	0.160	5.00	0.074	5.00	0.146	5.00	0.13 ± 0.10	5.00 ± 1.64
					78.3	0.028	5.00	0.019	5.00	-0.004	5.00	0.061	5.00	0.03 ± 0.03	5.00 ± 0.00
					19.6	0.036	2.90	0.001	5.00	-0.003	5.00	0.046	3.80	0.01 ± 0.02	4.60 ± 1.02
				250	5000	0.622	5.00	0.447	5.00	0.907	2.15	0.855	2.74	0.74 ± 0.21	3.30 ± 1.49
					1250	0.440	3.30	0.324	4.30	0.655	2.26	0.363	5.00	0.45 ± 0.15	3.85 ± 1.19
					313	0.334	1.50	0.160	5.00	0.100	5.00	0.126	5.00	0.13 ± 0.11	5.00 ± 1.75
					78.3	0.038	5.00	-0.014	5.00	-0.008	5.00	0.019	5.00	0.00 ± 0.02	5.00 ± 0.00
					19.6	0.030	3.34	-0.022	5.00	0.012	5.00	0.003	5.00	0.00 ± 0.02	5.00 ± 0.83
				125	5000	0.619	5.00	0.469	5.00	0.910	2.07	0.861	2.59	0.75 ± 0.21	3.22 ± 1.56
					1250	0.479	2.69	0.336	3.86	0.684	2.00	0.427	5.00	0.48 ± 0.15	3.62 ± 1.32
					313	0.323	1.57	0.138	5.00	0.106	5.00	0.135	5.00	0.13 ± 0.10	5.00 ± 1.72
					78.3	0.036	5.00	0.252	0.56	-0.007	5.00	0.036	5.00	0.09 ± 0.12	3.52 ± 2.22
					19.6	0.024	4.19	-0.026	5.00	0.010	5.00	0.002	5.00	0.00 ± 0.02	5.00 ± 0.41

Agents		Mechanism		Conc (ng/ml)		RD		SMS-CTR		RH30		RH41		Average	
Tx1	Tx2	Tx1	Tx2	Tx1	Tx2	FA	CI	FA	CI	FA	CI	FA	CI	FA	CI
Cabozantinib	4HC	TKI	Alkylator	2000	5000	0.993	1.18	1.000	0.03	1.000	5.00	1.000	0.44	1.00 ± 0.00	1.82 ± 2.27
					2500	0.383	2.37	1.000	0.02	0.997	0.56	0.927	0.86	0.97 ± 0.30	0.48 ± 1.01
					1250	0.337	1.47	0.501	1.86	0.431	1.40	0.286	1.44	0.41 ± 0.10	1.57 ± 0.21
					625	0.209	1.23	0.367	1.45	0.142	3.04	0.283	1.09	0.26 ± 0.10	1.86 ± 0.90
					313	0.150	1.13	0.222	1.39	0.119	3.33	0.299	0.85	0.21 ± 0.08	1.86 ± 1.12
				1000	5000	0.965	1.71	1.000	5.00	1.000	5.00	1.000	5.00	1.00 ± 0.02	5.00 ± 1.65
					2500	0.359	2.23	1.000	0.02	0.968	0.82	0.907	0.89	0.96 ± 0.30	0.58 ± 0.92
					1250	0.301	1.31	0.439	2.10	0.256	1.50	0.133	1.82	0.28 ± 0.13	1.81 ± 0.35
					625	0.183	0.97	0.364	1.35	0.035	5.00	0.104	1.76	0.17 ± 0.14	2.70 ± 1.85
					313	0.056	1.17	0.247	1.07	0.030	5.00	0.126	1.26	0.13 ± 0.10	2.44 ± 1.92
				500	5000	0.982	1.46	1.000	0.01	1.000	5.00	1.000	0.62	1.00 ± 0.01	1.88 ± 2.23
					2500	0.421	2.00	1.000	0.02	0.942	0.91	0.848	0.97	0.93 ± 0.26	0.63 ± 0.81
					1250	0.277	1.23	0.408	2.23	0.183	1.38	0.059	2.23	0.22 ± 0.15	1.95 ± 0.54
					625	0.163	0.83	0.378	1.24	0.001	5.00	0.048	2.06	0.14 ± 0.17	2.77 ± 1.88
					313	0.061	0.77	0.359	0.68	-0.001	5.00	0.024	3.65	0.13 ± 0.17	3.11 ± 2.15
				250	5000	0.828	2.52	1.000	5.00	1.000	5.00	1.000	5.00	1.00 ± 0.09	5.00 ± 1.24
					2500	0.340	2.12	1.000	0.02	0.929	0.93	0.872	0.94	0.93 ± 0.30	0.63 ± 0.86
					1250	0.313	1.12	0.493	1.76	0.195	1.11	0.032	2.32	0.24 ± 0.19	1.73 ± 0.58
					625	0.176	0.72	0.282	1.60	-0.004	5.00	0.014	3.56	0.10 ± 0.14	3.39 ± 1.93
					313	0.082	0.53	0.130	1.55	-0.018	5.00	-0.019	5.00	0.03 ± 0.07	3.85 ± 2.32
				125	5000	0.880	2.29	1.000	5.00	1.000	0.68	1.000	0.69	1.00 ± 0.06	2.12 ± 2.04
					2500	0.360	2.05	0.999	0.03	0.925	0.94	0.762	1.05	0.90 ± 0.29	0.67 ± 0.83
					1250	0.310	1.09	0.388	2.31	0.180	1.02	-0.005	5.00	0.19 ± 0.17	2.78 ± 1.86
					625	0.141	0.72	0.279	1.60	-0.017	5.00	0.009	3.14	0.09 ± 0.14	3.25 ± 1.88
					313	0.065	0.49	0.152	1.33	-0.025	5.00	-0.048	5.00	0.03 ± 0.09	3.78 ± 2.39
Cabozantinib	Bortezomib	TKI	Proteosome	2000	10	0.103	1.89	0.386	1.10	0.112	4.60	0.282	1.88	0.26 ± 0.14	2.53 ± 1.53
					6.67	0.093	1.68	0.217	1.21	0.101	4.54	0.287	1.47	0.20 ± 0.09	2.41 ± 1.56
					4.45	0.118	1.31	0.152	1.23	0.080	5.00	0.265	1.33	0.17 ± 0.08	2.52 ± 1.86
					2.97	0.072	1.57	0.139	1.09	0.112	3.66	0.268	1.14	0.17 ± 0.08	1.96 ± 1.22
					1.98	0.079	1.40	0.130	1.01	0.097	4.08	0.265	1.03	0.16 ± 0.08	2.04 ± 1.48
				1000	10	0.044	1.94	0.323	1.10	0.026	5.00	0.105	3.03	0.15 ± 0.14	3.04 ± 1.69
					6.67	0.026	2.00	0.122	1.37	0.010	5.00	0.072	3.34	0.07 ± 0.05	3.24 ± 1.61
					4.45	0.070	1.08	0.000	5.00	0.019	5.00	0.093	2.31	0.04 ± 0.04	4.10 ± 1.97
					2.97	0.005	3.84	0.019	2.78	0.003	5.00	0.084	2.25	0.04 ± 0.04	3.34 ± 1.22
					1.98	0.027	1.40	0.109	0.73	0.019	5.00	0.075	2.32	0.07 ± 0.04	2.68 ± 1.88
				500	10	0.046	1.47	0.248	1.19	0.009	5.00	0.018	5.00	0.09 ± 0.11	3.73 ± 2.12
					6.67	0.034	1.27	0.041	2.00	0.021	5.00	0.007	5.00	0.02 ± 0.01	4.00 ± 1.97
					4.45	0.005	2.34	-0.012	5.00	-0.001	5.00	-0.007	5.00	-0.01 ± 0.01	5.00 ± 1.33
					2.97	0.022	1.04	-0.044	5.00	0.014	5.00	0.001	5.00	-0.01 ± 0.03	5.00 ± 1.98
					1.98	0.023	0.89	0.086	0.60	0.024	3.93	0.015	5.00	0.04 ± 0.03	3.18 ± 2.19
				250	10	0.047	1.25	0.248	1.14	0.009	5.00	-0.027	5.00	0.08 ± 0.12	3.71 ± 2.20
					6.67	0.039	0.96	0.016	2.67	0.000	5.00	-0.037	5.00	-0.01 ± 0.03	4.22 ± 1.97
					4.45	0.015	1.02	0.006	3.27	0.008	5.00	-0.039	5.00	-0.01 ± 0.02	4.42 ± 1.89
					2.97	0.029	0.63	0.025	1.18	0.010	4.80	-0.030	5.00	0.00 ± 0.03	3.66 ± 2.32
					1.98	0.016	0.67	-0.043	5.00	0.011	3.99	-0.041	5.00	-0.02 ± 0.03	4.66 ± 2.05
				125	10	0.057	1.09	0.194	1.26	0.003	5.00	-0.018	5.00	0.06 ± 0.10	3.75 ± 2.21
					6.67	0.057	0.76	0.089	1.19	0.020	2.50	-0.050	5.00	0.02 ± 0.06	2.90 ± 1.91
					4.45	0.036	0.62	-0.077	5.00	0.004	5.00	-0.057	5.00	-0.04 ± 0.05	5.00 ± 2.19
					2.97	0.038	0.45	0.095	0.55	0.016	1.95	-0.049	5.00	0.02 ± 0.06	2.50 ± 2.12
					1.98	0.049	0.31	-0.056	5.00	0.016	1.78	-0.043	5.00	-0.03 ± 0.05	3.93 ± 2.36
Cabozantinib	SN-38	TKI	Topo I	2000	5	0.571	1.32	0.401	0.96	0.853	0.51	0.571	0.76	0.61 ± 0.19	0.74 ± 0.34
					2.5	0.508	1.03	0.343	0.75	0.794	0.45	0.425	0.82	0.52 ± 0.20	0.67 ± 0.24
					1.25	0.472	0.76	0.320	0.56	0.724	0.44	0.332	0.88	0.46 ± 0.19	0.62 ± 0.20
					0.625	0.420	0.65	0.276	0.51	0.558	0.65	0.297	0.85	0.38 ± 0.13	0.67 ± 0.14
					0.313	0.288	0.80	0.235	0.51	0.232	1.97	0.301	0.75	0.26 ± 0.04	1.08 ± 0.65
				1000	5	0.539	1.39	0.376	0.95	0.854	0.45	0.454	0.97	0.56 ± 0.21	0.79 ± 0.39
					2.5	0.475	1.03	0.342	0.63	0.790	0.38	0.263	1.19	0.47 ± 0.23	0.73 ± 0.37
					1.25	0.449	0.67	0.303	0.45	0.688	0.39	0.189	1.18	0.39 ± 0.22	0.67 ± 0.36
					0.625	0.395	0.52	0.268	0.36	0.417	0.75	0.164	1.07	0.28 ± 0.12	0.73 ± 0.31
					0.313	0.231	0.74	0.168	0.47	0.083	3.78	0.127	1.24	0.13 ± 0.06	1.83 ± 1.52
				500	5	0.544	1.30	0.393	0.83	0.854	0.42	0.423	0.98	0.56 ± 0.21	0.74 ± 0.36
					2.5	0.472	0.96	0.302	0.69	0.780	0.37	0.238	1.10	0.44 ± 0.24	0.72 ± 0.32
					1.25	0.409	0.71	0.280	0.43	0.666	0.36	0.091	1.83	0.35 ± 0.24	0.87 ± 0.68
					0.625	0.372	0.48	0.283	0.25	0.348	0.76	0.051	2.35	0.23 ± 0.15	1.12 ± 0.95
					0.313	0.211	0.68	0.150	0.37	0.058	3.85	0.042	2.36	0.08 ± 0.08	2.19 ± 1.61
				250	5	0.536	1.31	0.385	0.83	0.851	0.42	0.416	0.96	0.55 ± 0.21	0.73 ± 0.37
					2.5	0.451	1.03	0.339	0.54	0.779	0.35	0.232	1.02	0.45 ± 0.24	0.64 ± 0.35
					1.25	0.415	0.64	0.299	0.35	0.624	0.39	0.096	1.38	0.34 ± 0.22	0.71 ± 0.48
					0.625	0.394	0.38	0.274	0.22	0.279	0.88	0.048	1.72	0.20 ± 0.15	0.94 ± 0.67
					0.313	0.199	0.66	0.137	0.33	0.021	5.00	0.014	4.11	0.06 ± 0.09	3.15 ± 2.38
				125	5	0.528	1.35	0.374	0.86	0.839	0.45	0.412	0.95	0.54 ± 0.21	0.75 ± 0.37
					2.5	0.461	0.96	0.319	0.58	0.765	0.36	0.200	1.11	0.43 ± 0.24	0.68 ± 0.34
					1.25	0.412	0.63	0.269	0.39	0.621	0.37	0.122	0.96	0.34 ± 0.21	0.57 ± 0.28
					0.625	0.371	0.40	0.352	0.13	0.288	0.78	0.040	1.54	0.23 ± 0.15	0.82 ± 0.61
					0.313	0.218	0.53	0.099	0.43	-0.002	5.00	0.008	4.71	0.03 ± 0.10	3.38 ± 2.53

Agents Tx1	Agents Tx2	Mechanism Tx1	Mechanism Tx2	Conc (ng/ml) Tx1	Conc (ng/ml) Tx2	RD FA	RD CI	SMS-CTR FA	SMS-CTR CI	RH30 FA	RH30 CI	RH41 FA	RH41 CI	Average FA	Average CI
Cabozantinib	Vinorelbine	TKI	Microtubule	2000	4	0.647	3.92	0.804	2.10	0.584	0.46	0.853	0.80	0.75 ± 0.13	1.12 ± 1.57
					2	0.431	4.80	0.635	2.38	0.382	0.99	0.735	0.87	0.58 ± 0.17	1.42 ± 1.83
					1	0.270	5.00	0.411	2.89	0.238	1.86	0.495	1.36	0.38 ± 0.12	2.04 ± 1.61
					0.5	0.149	5.00	0.273	2.75	0.158	2.88	0.387	1.28	0.27 ± 0.11	2.30 ± 1.53
					0.25	0.147	3.22	0.285	1.46	0.145	2.88	0.331	1.10	0.25 ± 0.10	1.81 ± 1.04
				1000	4	0.731	2.60	0.822	1.85	0.557	0.30	0.783	1.24	0.72 ± 0.12	1.13 ± 0.97
					2	0.390	5.00	0.605	2.63	0.240	1.51	0.421	3.11	0.42 ± 0.15	2.42 ± 1.46
					1	0.146	5.00	0.294	4.51	0.097	5.00	0.219	4.24	0.20 ± 0.09	4.58 ± 0.38
					0.5	0.072	5.00	0.213	3.49	0.044	5.00	0.148	3.85	0.13 ± 0.08	4.11 ± 0.78
					0.25	0.045	5.00	0.104	4.04	0.041	5.00	0.139	2.53	0.09 ± 0.05	3.86 ± 1.17
				500	4	0.768	2.11	0.829	1.76	0.563	0.19	0.722	1.69	0.70 ± 0.11	1.21 ± 0.85
					2	0.382	5.00	0.620	2.45	0.194	1.86	0.299	5.00	0.37 ± 0.18	3.10 ± 1.66
					1	0.124	5.00	0.262	5.00	0.056	5.00	0.122	5.00	0.15 ± 0.09	5.00 ± 0.00
					0.5	0.048	5.00	0.077	5.00	0.035	5.00	0.062	5.00	0.06 ± 0.02	5.00 ± 0.00
					0.25	0.032	5.00	0.055	5.00	0.040	5.00	0.058	5.00	0.05 ± 0.01	5.00 ± 0.00
				250	4	0.784	1.91	0.831	1.73	0.545	0.16	0.683	2.01	0.69 ± 0.13	1.30 ± 0.87
					2	0.370	5.00	0.615	2.49	0.157	2.65	0.213	5.00	0.33 ± 0.21	3.38 ± 1.40
					1	0.108	5.00	0.203	5.00	0.038	5.00	0.066	5.00	0.10 ± 0.07	5.00 ± 0.00
					0.5	0.046	5.00	0.077	5.00	0.008	5.00	0.020	5.00	0.04 ± 0.03	5.00 ± 0.00
					0.25	-0.001	5.00	-0.008	5.00	0.014	5.00	0.025	5.00	0.01 ± 0.02	5.00 ± 0.00
				125	4	0.779	1.96	0.829	1.75	0.527	0.15	0.692	1.93	0.68 ± 0.13	1.28 ± 0.87
					2	0.330	5.00	0.625	2.38	0.142	3.18	0.184	5.00	0.32 ± 0.22	3.52 ± 1.32
					1	0.091	5.00	0.137	5.00	0.025	5.00	0.062	5.00	0.07 ± 0.05	5.00 ± 0.00
					0.5	0.019	5.00	0.059	5.00	0.004	5.00	0.004	5.00	0.02 ± 0.03	5.00 ± 0.00
					0.25	0.023	5.00	0.039	5.00	0.032	5.00	-0.001	5.00	0.02 ± 0.02	5.00 ± 0.00
Etoposide	Actinomycin D	Topo II	Alkylator	5000	2	0.779	5.00	0.545	5.00	0.887	2.61	0.921	1.96	0.78 ± 0.17	3.19 ± 1.59
					1	0.707	5.00	0.525	5.00	0.866	3.07	0.908	1.93	0.77 ± 0.17	3.33 ± 1.52
					0.5	0.692	5.00	0.540	3.75	0.861	3.18	0.892	2.08	0.76 ± 0.16	3.00 ± 1.22
					0.25	0.647	5.00	0.510	4.25	0.855	3.30	0.885	2.14	0.75 ± 0.18	3.23 ± 1.24
					0.125	0.647	5.00	0.582	2.24	0.856	3.29	0.867	2.50	0.77 ± 0.15	2.68 ± 1.24
				1250	2	0.679	5.00	0.445	5.00	0.614	2.89	0.751	3.02	0.60 ± 0.13	3.64 ± 1.18
					1	0.604	5.00	0.430	5.00	0.566	3.29	0.596	4.41	0.53 ± 0.08	4.23 ± 0.81
					0.5	0.549	5.00	0.388	5.00	0.566	3.20	0.560	4.41	0.50 ± 0.09	4.20 ± 0.85
					0.25	0.503	5.00	0.378	4.65	0.560	3.21	0.545	4.33	0.49 ± 0.08	4.06 ± 0.77
					0.125	0.486	5.00	0.389	3.32	0.594	2.81	0.483	5.00	0.49 ± 0.08	3.71 ± 1.14
				313	2	0.620	5.00	0.520	4.00	0.251	5.00	0.471	4.85	0.41 ± 0.16	4.62 ± 0.48
					1	0.562	5.00	0.372	5.00	0.184	5.00	0.277	5.00	0.28 ± 0.16	5.00 ± 0.00
					0.5	0.427	5.00	0.237	5.00	0.081	5.00	0.191	5.00	0.17 ± 0.14	5.00 ± 0.00
					0.25	0.299	5.00	0.177	5.00	-0.014	5.00	0.217	5.00	0.13 ± 0.13	5.00 ± 0.00
					0.125	0.233	5.00	0.164	5.00	-0.016	5.00	0.182	5.00	0.11 ± 0.11	5.00 ± 0.00
				78.3	2	0.590	5.00	0.476	5.00	0.134	5.00	0.398	4.66	0.34 ± 0.19	4.89 ± 0.17
					1	0.518	5.00	0.438	4.37	0.035	5.00	0.205	5.00	0.23 ± 0.22	4.79 ± 0.32
					0.5	0.309	5.00	0.199	5.00	-0.092	5.00	0.108	5.00	0.07 ± 0.17	5.00 ± 0.00
					0.25	0.106	5.00	0.091	5.00	-0.036	5.00	0.080	5.00	0.04 ± 0.07	5.00 ± 0.00
					0.125	0.072	5.00	0.178	5.00	0.007	5.00	0.050	5.00	0.08 ± 0.07	5.00 ± 0.00
				19.6	2	0.577	5.00	0.437	5.00	0.138	5.00	0.330	5.00	0.30 ± 0.19	5.00 ± 0.00
					1	0.463	5.00	0.370	5.00	0.041	5.00	0.179	4.64	0.20 ± 0.19	4.88 ± 0.18
					0.5	0.274	5.00	0.150	5.00	-0.075	5.00	0.075	5.00	0.05 ± 0.15	5.00 ± 0.00
					0.25	0.081	5.00	0.066	5.00	-0.034	5.00	0.025	5.00	0.02 ± 0.05	5.00 ± 0.00
					0.125	0.050	5.00	0.036	5.00	0.004	5.00	0.019	5.00	0.02 ± 0.02	5.00 ± 0.00
SN-38	Actinomycin D	Topo I	Alkylator	5	2	0.765	5.00	0.472	5.00	0.825	0.54	0.733	2.01	0.68 ± 0.16	2.52 ± 2.23
					1	0.693	5.00	0.454	4.21	0.804	0.59	0.633	1.59	0.63 ± 0.15	2.13 ± 2.10
					0.5	0.649	4.44	0.469	2.11	0.809	0.56	0.591	1.16	0.62 ± 0.14	1.28 ± 1.71
					0.25	0.585	3.74	0.452	1.51	0.802	0.58	0.547	0.96	0.60 ± 0.15	1.01 ± 1.42
					0.125	0.550	2.87	0.465	0.96	0.824	0.49	0.584	0.70	0.62 ± 0.15	0.72 ± 1.09
				2.5	2	0.704	5.00	0.463	5.00	0.730	0.55	0.600	2.74	0.60 ± 0.12	2.76 ± 2.13
					1	0.657	5.00	0.426	5.00	0.703	0.56	0.448	2.24	0.53 ± 0.14	2.60 ± 2.19
					0.5	0.571	5.00	0.403	3.40	0.724	0.47	0.372	1.65	0.50 ± 0.16	1.84 ± 1.99
					0.25	0.508	5.00	0.392	2.09	0.725	0.45	0.315	1.30	0.48 ± 0.18	1.28 ± 1.98
					0.125	0.465	3.67	0.425	0.94	0.735	0.42	0.305	1.01	0.49 ± 0.18	0.79 ± 1.46
				1.25	2	0.696	5.00	0.434	5.00	0.602	0.71	0.428	4.09	0.49 ± 0.13	3.27 ± 2.04
					1	0.629	5.00	0.388	5.00	0.581	0.60	0.279	3.30	0.42 ± 0.16	2.97 ± 2.08
					0.5	0.543	5.00	0.351	5.00	0.595	0.48	0.190	2.56	0.38 ± 0.19	2.68 ± 2.18
					0.25	0.500	4.87	0.362	2.56	0.581	0.46	0.147	1.92	0.36 ± 0.19	1.65 ± 1.84
					0.125	0.421	4.14	0.388	1.08	0.597	0.41	0.150	1.29	0.38 ± 0.18	0.93 ± 1.65
				0.625	2	0.665	5.00	0.425	5.00	0.419	1.52	0.348	4.94	0.40 ± 0.14	3.82 ± 1.73
					1	0.616	5.00	0.552	1.43	0.403	1.05	0.180	4.49	0.38 ± 0.19	2.32 ± 2.04
					0.5	0.490	5.00	0.333	5.00	0.335	1.08	0.077	4.50	0.25 ± 0.17	3.53 ± 1.89
					0.25	0.421	5.00	0.345	2.92	0.291	1.06	0.069	2.77	0.23 ± 0.15	2.25 ± 1.61
					0.125	0.389	4.63	0.457	0.51	0.279	0.94	0.075	1.61	0.27 ± 0.17	1.02 ± 1.86
				0.313	2	0.622	5.00	0.550	2.85	0.262	4.15	0.298	5.00	0.37 ± 0.18	4.00 ± 1.02
					1	0.577	5.00	0.429	4.73	0.218	3.32	0.151	4.97	0.27 ± 0.20	4.34 ± 0.80
					0.5	0.417	5.00	0.271	5.00	0.070	5.00	0.059	5.00	0.13 ± 0.17	5.00 ± 0.00
					0.25	0.341	5.00	0.261	5.00	-0.010	5.00	0.004	5.00	0.08 ± 0.18	5.00 ± 0.00
					0.125	0.381	4.67	0.369	1.14	0.025	5.00	0.055	1.63	0.15 ± 0.19	2.59 ± 2.01

Agents Tx1	Agents Tx2	Mechanism Tx1	Mechanism Tx2	Conc (ng/ml) Tx1	Conc (ng/ml) Tx2	RD FA	RD CI	SMS-CTR FA	SMS-CTR CI	RH30 FA	RH30 CI	RH41 FA	RH41 CI	Average FA	Average CI
Etoposide	4HC	Topo II	Alkylator	5000	5000	0.903	2.73	0.999	0.09	0.999	1.00	0.998	1.00	1.00 ± 0.05	0.70 ± 1.10
					2500	0.592	5.00	0.998	0.06	0.959	1.84	0.982	0.91	0.98 ± 0.19	0.94 ± 2.16
					1250	0.617	5.00	0.622	2.76	0.895	2.90	0.841	3.55	0.79 ± 0.15	3.07 ± 1.02
					625	0.625	5.00	0.549	3.51	0.878	3.04	0.855	2.97	0.76 ± 0.16	3.17 ± 0.94
					313	0.637	4.90	0.494	4.69	0.870	3.09	0.856	2.81	0.74 ± 0.18	3.53 ± 1.07
				1250	5000	0.910	2.25	0.999	0.09	0.996	1.17	0.998	0.96	1.00 ± 0.04	0.74 ± 0.89
					2500	0.458	4.82	0.999	0.05	0.933	1.31	0.927	1.13	0.95 ± 0.25	0.83 ± 2.07
					1250	0.455	3.95	0.469	3.14	0.679	2.64	0.363	5.00	0.50 ± 0.13	3.59 ± 1.03
					625	0.445	3.64	0.396	3.44	0.658	2.52	0.416	5.00	0.49 ± 0.12	3.65 ± 1.02
					313	0.491	2.74	0.378	3.26	0.600	2.90	0.421	5.00	0.47 ± 0.10	3.72 ± 1.04
				313	5000	0.877	2.34	0.999	0.09	0.999	0.94	0.998	0.96	1.00 ± 0.06	0.66 ± 0.93
					2500	0.434	2.73	0.998	0.05	0.756	1.53	0.928	0.91	0.89 ± 0.25	0.83 ± 1.13
					1250	0.401	1.98	0.590	1.48	0.305	2.80	0.132	5.00	0.34 ± 0.19	3.09 ± 1.55
					625	0.358	1.77	0.349	2.14	0.135	5.00	0.150	5.00	0.21 ± 0.12	4.05 ± 1.76
					313	0.350	1.57	0.413	1.04	0.050	5.00	0.150	5.00	0.20 ± 0.17	3.68 ± 2.14
				78.3	5000	0.884	2.27	0.999	0.09	0.999	0.95	0.998	0.96	1.00 ± 0.06	0.66 ± 0.90
					2500	0.404	2.19	0.998	0.05	0.827	1.15	0.888	0.94	0.90 ± 0.26	0.71 ± 0.88
					1250	0.359	1.33	0.426	2.19	0.114	2.45	0.082	5.00	0.21 ± 0.17	3.21 ± 1.58
					625	0.185	1.60	0.301	1.81	0.026	5.00	0.078	5.00	0.13 ± 0.12	3.94 ± 1.90
					313	0.095	2.97	0.208	1.88	0.018	5.00	0.052	5.00	0.09 ± 0.08	3.96 ± 1.55
				19.6	5000	0.852	2.41	0.999	0.08	0.999	0.97	0.998	0.96	1.00 ± 0.07	0.67 ± 0.96
					2500	0.400	2.01	0.998	0.05	0.676	1.28	0.733	1.10	0.80 ± 0.25	0.81 ± 0.81
					1250	0.327	1.14	0.481	1.82	0.158	1.18	0.089	1.90	0.24 ± 0.18	1.63 ± 0.41
					625	0.161	0.95	0.307	1.52	0.035	1.69	0.080	1.59	0.14 ± 0.12	1.60 ± 0.33
					313	0.096	1.01	0.180	1.45	0.057	0.99	0.027	4.26	0.09 ± 0.07	2.23 ± 1.57
SN-38	4HC	Topo I	Alkylator	5	5000	0.996	1.01	1.000	5.00	1.000	0.64	1.000	0.65	1.00 ± 0.00	2.10 ± 2.12
					2500	0.652	2.23	0.987	0.21	0.988	0.72	0.895	1.03	0.96 ± 0.16	0.65 ± 0.86
					1250	0.579	1.85	0.602	1.60	0.860	0.90	0.469	1.41	0.64 ± 0.17	1.30 ± 0.40
					625	0.576	1.45	0.544	1.15	0.849	0.68	0.460	1.11	0.62 ± 0.17	0.98 ± 0.32
					313	0.560	1.34	0.544	0.76	0.843	0.56	0.476	0.91	0.62 ± 0.16	0.75 ± 0.33
				2.5	5000	0.994	1.15	1.000	5.00	1.000	5.00	1.000	0.69	1.00 ± 0.00	3.56 ± 2.36
					2500	0.547	2.31	1.000	5.00	0.944	0.96	0.868	1.01	0.94 ± 0.20	2.32 ± 1.89
					1250	0.484	1.74	0.571	1.59	0.780	0.90	0.295	1.41	0.55 ± 0.20	1.30 ± 0.37
					625	0.459	1.41	0.524	1.01	0.755	0.66	0.291	1.06	0.52 ± 0.19	0.91 ± 0.31
					313	0.476	1.10	0.434	0.83	0.759	0.51	0.267	0.96	0.49 ± 0.20	0.76 ± 0.25
				1.25	5000	0.982	1.46	1.000	5.00	1.000	0.59	1.000	0.64	1.00 ± 0.01	2.07 ± 2.09
					2500	0.467	2.29	1.000	5.00	0.936	0.95	0.759	1.12	0.90 ± 0.24	2.36 ± 1.87
					1250	0.404	1.60	0.575	1.49	0.682	0.89	0.157	1.47	0.47 ± 0.23	1.28 ± 0.32
					625	0.388	1.18	0.475	1.04	0.636	0.65	0.111	1.33	0.41 ± 0.22	1.01 ± 0.29
					313	0.418	0.83	0.545	0.48	0.617	0.52	0.125	1.02	0.43 ± 0.22	0.67 ± 0.26
				0.625	5000	0.958	1.77	1.000	5.00	1.000	5.00	1.000	0.71	1.00 ± 0.02	3.57 ± 2.21
					2500	0.393	2.30	0.999	0.04	0.938	0.93	0.633	1.21	0.86 ± 0.28	0.72 ± 0.93
					1250	0.372	1.38	0.475	1.89	0.535	0.94	0.069	1.62	0.36 ± 0.21	1.48 ± 0.41
					625	0.399	0.81	0.481	0.96	0.424	0.76	0.064	1.20	0.32 ± 0.19	0.97 ± 0.20
					313	0.383	0.60	0.346	0.77	0.359	0.70	0.052	1.10	0.25 ± 0.16	0.86 ± 0.22
				0.313	5000	0.992	1.20	1.000	5.00	1.000	5.00	0.999	0.77	1.00 ± 0.00	3.59 ± 2.33
					2500	0.456	1.97	0.992	0.16	0.979	0.76	0.751	1.07	0.91 ± 0.25	0.66 ± 0.76
					1250	0.475	1.02	0.473	1.87	0.379	0.99	0.025	1.91	0.29 ± 0.21	1.59 ± 0.51
					625	0.352	0.72	0.423	1.09	0.189	1.05	0.029	1.24	0.21 ± 0.18	1.13 ± 0.22
					313	0.373	0.44	0.355	0.69	0.080	1.88	0.023	1.11	0.15 ± 0.18	1.23 ± 0.63
SN-38	Etoposide	Topo I	Topo II	5	5000	0.802	1.88	0.644	1.51	0.960	1.06	0.921	1.39	0.84 ± 0.14	1.32 ± 0.34
					1250	0.630	2.00	0.499	1.49	0.873	1.06	0.684	2.44	0.69 ± 0.15	1.66 ± 0.60
					313	0.554	1.62	0.428	1.10	0.878	0.49	0.479	2.08	0.60 ± 0.20	1.22 ± 0.68
					78.3	0.535	1.41	0.428	0.76	0.881	0.36	0.440	1.24	0.58 ± 0.21	0.79 ± 0.48
					19.6	0.523	1.40	0.435	0.66	0.873	0.35	0.426	0.98	0.58 ± 0.21	0.66 ± 0.45
				2.5	5000	0.728	2.98	0.573	2.44	0.941	1.47	0.902	1.72	0.81 ± 0.17	1.88 ± 0.69
					1250	0.560	2.33	0.427	2.13	0.816	1.32	0.587	3.55	0.61 ± 0.16	2.33 ± 0.92
					313	0.481	1.51	0.377	1.09	0.798	0.59	0.328	3.31	0.50 ± 0.21	1.66 ± 1.19
					78.3	0.468	1.08	0.429	0.44	0.782	0.40	0.245	1.91	0.49 ± 0.22	0.92 ± 0.71
					19.6	0.435	1.13	0.375	0.46	0.794	0.32	0.221	1.25	0.46 ± 0.24	0.68 ± 0.47
				1.25	5000	0.688	3.68	0.595	1.98	0.919	1.92	0.885	2.06	0.80 ± 0.16	1.99 ± 0.85
					1250	0.495	2.85	0.374	2.99	0.769	1.54	0.519	4.62	0.55 ± 0.17	3.05 ± 1.26
					313	0.424	1.46	0.354	1.06	0.675	0.79	0.198	5.00	0.41 ± 0.20	2.28 ± 1.97
					78.3	0.423	0.80	0.339	0.49	0.599	0.56	0.108	4.05	0.35 ± 0.20	1.70 ± 1.72
					19.6	0.389	0.75	0.328	0.33	0.623	0.39	0.076	2.45	0.34 ± 0.22	1.06 ± 1.00
				0.625	5000	0.656	4.34	0.491	4.39	0.913	2.03	0.871	2.36	0.76 ± 0.20	2.93 ± 1.26
					1250	0.495	2.67	0.369	2.99	0.730	1.74	0.565	3.70	0.55 ± 0.15	2.81 ± 0.81
					313	0.403	1.31	0.304	1.43	0.482	1.35	0.168	5.00	0.32 ± 0.14	2.59 ± 1.82
					78.3	0.390	0.61	0.342	0.35	0.314	1.10	0.047	5.00	0.23 ± 0.15	2.15 ± 2.18
					19.6	0.374	0.44	0.274	0.29	0.241	1.06	0.071	1.96	0.20 ± 0.13	1.10 ± 0.76
				0.313	5000	0.650	4.43	0.494	4.27	0.912	2.03	0.874	2.29	0.76 ± 0.20	2.86 ± 1.27
					1250	0.459	3.08	0.357	3.27	0.718	1.78	0.444	5.00	0.51 ± 0.16	3.35 ± 1.32
					313	0.402	1.15	0.432	0.47	0.313	2.25	0.140	5.00	0.29 ± 0.13	2.57 ± 1.99
					78.3	0.280	0.82	0.246	0.68	0.071	4.13	0.046	5.00	0.12 ± 0.12	3.27 ± 2.23
					19.6	0.219	0.68	0.356	0.11	0.076	2.25	0.005	5.00	0.15 ± 0.16	2.45 ± 2.19

Agents Tx1	Agents Tx2	Mechanism Tx1	Mechanism Tx2	Conc (ng/ml) Tx1	Conc (ng/ml) Tx2	RD FA	RD CI	SMS-CTR FA	SMS-CTR CI	RH30 FA	RH30 CI	RH41 FA	RH41 CI	Average FA	Average CI
SN-38	Vinorelbine	Topo I	Microtubule	5	4	**0.548**	*5.00*	**0.612**	*5.00*	**0.870**	*0.35*	**0.568**	*3.81*	**0.68** ± 0.15	*3.05 ± 2.20*
					2	**0.525**	*4.42*	**0.526**	*3.90*	**0.858**	*0.38*	**0.514**	*2.68*	**0.63** ± 0.17	*2.32 ± 1.80*
					1	**0.513**	*3.04*	**0.477**	*2.62*	**0.856**	*0.39*	**0.478**	*1.91*	**0.60** ± 0.18	*1.64 ± 1.16*
					0.5	**0.504**	*2.34*	**0.455**	*1.72*	**0.838**	*0.45*	**0.473**	*1.35*	**0.59** ± 0.18	*1.17 ± 0.79*
					0.25	**0.497**	*1.99*	**0.526**	*0.85*	**0.863**	*0.37*	**0.466**	*1.08*	**0.62** ± 0.18	*0.77 ± 0.68*
				2.5	4	**0.537**	*5.00*	**0.590**	*5.00*	**0.804**	*0.29*	**0.439**	*5.00*	**0.61** ± 0.15	*3.43 ± 2.36*
					2	**0.467**	*4.78*	**0.499**	*4.09*	**0.794**	*0.30*	**0.316**	*5.00*	**0.54** ± 0.20	*3.13 ± 2.19*
					1	**0.445**	*3.12*	**0.434**	*2.78*	**0.764**	*0.36*	**0.266**	*3.67*	**0.49** ± 0.21	*2.27 ± 1.46*
					0.5	**0.446**	*2.06*	**0.385**	*1.89*	**0.760**	*0.36*	**0.275**	*2.12*	**0.47** ± 0.21	*1.46 ± 0.84*
					0.25	**0.441**	*1.58*	**0.388**	*1.13*	**0.789**	*0.31*	**0.244**	*1.66*	**0.47** ± 0.23	*1.03 ± 0.62*
				1.25	4	**0.529**	*5.00*	**0.605**	*5.00*	**0.702**	*0.27*	**0.351**	*5.00*	**0.55** ± 0.15	*3.42 ± 2.37*
					2	**0.433**	*4.96*	**0.506**	*3.87*	**0.663**	*0.31*	**0.195**	*5.00*	**0.45** ± 0.19	*3.06 ± 2.21*
					1	**0.401**	*3.16*	**0.412**	*2.85*	**0.618**	*0.37*	**0.118**	*5.00*	**0.38** ± 0.21	*2.74 ± 1.90*
					0.5	**0.387**	*2.03*	**0.360**	*1.88*	**0.621**	*0.36*	**0.136**	*4.04*	**0.37** ± 0.20	*2.09 ± 1.51*
					0.25	**0.387**	*1.36*	**0.358**	*1.06*	**0.654**	*0.31*	**0.122**	*2.68*	**0.38** ± 0.22	*1.35 ± 0.99*
				0.625	4	**0.581**	*5.00*	**0.607**	*5.00*	**0.569**	*0.30*	**0.468**	*4.93*	**0.55** ± 0.06	*3.41 ± 2.34*
					2	**0.418**	*4.99*	**0.453**	*4.66*	**0.369**	*0.71*	**0.155**	*5.00*	**0.33** ± 0.13	*3.46 ± 2.09*
					1	**0.373**	*3.20*	**0.312**	*4.18*	**0.264**	*1.10*	**0.093**	*5.00*	**0.22** ± 0.12	*3.43 ± 1.68*
					0.5	**0.360**	*1.90*	**0.321**	*2.07*	**0.282**	*0.85*	**0.081**	*5.00*	**0.23** ± 0.12	*2.64 ± 1.78*
					0.25	**0.357**	*1.17*	**0.398**	*0.80*	**0.327**	*0.63*	**0.063**	*4.50*	**0.26** ± 0.15	*1.98 ± 1.83*
				0.313	4	**0.644**	*3.80*	**0.703**	*3.44*	**0.524**	*0.25*	**0.607**	*2.80*	**0.61** ± 0.08	*2.16 ± 1.60*
					2	**0.333**	*5.00*	**0.441**	*4.83*	**0.142**	*3.86*	**0.138**	*5.00*	**0.24** ± 0.15	*4.56 ± 0.55*
					1	**0.196**	*5.00*	**0.271**	*4.95*	**0.037**	*5.00*	**0.062**	*5.00*	**0.12** ± 0.11	*4.98 ± 0.02*
					0.5	**0.205**	*3.80*	**0.123**	*5.00*	**0.040**	*5.00*	**-0.002**	*5.00*	**0.05** ± 0.09	*5.00 ± 0.60*
					0.25	**0.193**	*2.35*	**0.353**	*0.91*	**0.069**	*3.89*	**0.002**	*5.00*	**0.14** ± 0.15	*3.27 ± 1.79*
Vinorelbine	Actinomycin D	Microtubule	Alkylator	4	2	**0.752**	*5.00*	**0.863**	*1.42*	**0.553**	*0.55*	**0.630**	*4.82*	**0.68** ± 0.14	*2.26 ± 2.29*
					1	**0.682**	*5.00*	**0.828**	*1.81*	**0.550**	*0.33*	**0.612**	*3.93*	**0.66** ± 0.12	*2.02 ± 2.10*
					0.5	**0.653**	*5.00*	**0.836**	*1.69*	**0.459**	*0.43*	**0.534**	*4.46*	**0.61** ± 0.16	*2.19 ± 2.19*
					0.25	**0.650**	*5.00*	**0.807**	*2.03*	**0.394**	*0.53*	**0.538**	*4.02*	**0.58** ± 0.17	*2.19 ± 2.00*
					0.125	**0.654**	*4.52*	**0.820**	*1.86*	**0.349**	*0.66*	**0.535**	*3.89*	**0.57** ± 0.20	*2.14 ± 1.79*
				2	2	**0.650**	*5.00*	**0.655**	*3.06*	**0.371**	*1.81*	**0.394**	*5.00*	**0.47** ± 0.16	*3.29 ± 1.57*
					1	**0.525**	*5.00*	**0.628**	*2.99*	**0.269**	*2.34*	**0.266**	*5.00*	**0.39** ± 0.18	*3.44 ± 1.37*
					0.5	**0.353**	*5.00*	**0.485**	*5.00*	**0.170**	*4.31*	**0.144**	*5.00*	**0.27** ± 0.16	*4.77 ± 0.35*
					0.25	**0.207**	*5.00*	**0.410**	*5.00*	**0.048**	*5.00*	**0.095**	*5.00*	**0.18** ± 0.16	*5.00 ± 0.00*
					0.125	**0.157**	*5.00*	**0.435**	*5.00*	**0.059**	*5.00*	**0.119**	*5.00*	**0.20** ± 0.17	*5.00 ± 0.00*
				1	2	**0.595**	*5.00*	**0.548**	*4.46*	**0.304**	*2.83*	**0.337**	*5.00*	**0.40** ± 0.15	*4.10 ± 1.03*
					1	**0.500**	*5.00*	**0.506**	*4.05*	**0.222**	*3.17*	**0.226**	*5.00*	**0.32** ± 0.16	*4.07 ± 0.88*
					0.5	**0.266**	*5.00*	**0.278**	*5.00*	**0.092**	*5.00*	**0.073**	*5.00*	**0.15** ± 0.11	*5.00 ± 0.00*
					0.25	**0.107**	*5.00*	**0.308**	*5.00*	**0.007**	*5.00*	**0.024**	*5.00*	**0.11** ± 0.14	*5.00 ± 0.00*
					0.125	**0.089**	*5.00*	**0.161**	*5.00*	**0.011**	*5.00*	**0.040**	*5.00*	**0.07** ± 0.07	*5.00 ± 0.00*
				0.5	2	**0.571**	*5.00*	**0.541**	*3.90*	**0.309**	*2.63*	**0.327**	*5.00*	**0.39** ± 0.14	*3.84 ± 1.13*
					1	**0.463**	*5.00*	**0.406**	*5.00*	**0.204**	*3.52*	**0.202**	*5.00*	**0.27** ± 0.14	*4.51 ± 0.74*
					0.5	**0.241**	*5.00*	**0.180**	*5.00*	**0.072**	*5.00*	**0.061**	*5.00*	**0.10** ± 0.09	*5.00 ± 0.00*
					0.25	**0.060**	*5.00*	**0.170**	*5.00*	**0.013**	*5.00*	**0.002**	*5.00*	**0.06** ± 0.08	*5.00 ± 0.00*
					0.125	**0.003**	*5.00*	**0.252**	*5.00*	**0.016**	*5.00*	**0.006**	*5.00*	**0.09** ± 0.12	*5.00 ± 0.00*
				0.25	2	**0.579**	*5.00*	**0.550**	*3.21*	**0.311**	*2.54*	**0.324**	*5.00*	**0.40** ± 0.14	*3.58 ± 1.26*
					1	**0.463**	*5.00*	**0.536**	*2.03*	**0.227**	*2.69*	**0.199**	*4.93*	**0.32** ± 0.17	*3.22 ± 1.53*
					0.5	**0.218**	*5.00*	**0.190**	*5.00*	**0.108**	*5.00*	**0.106**	*5.00*	**0.13** ± 0.06	*5.00 ± 0.00*
					0.25	**0.051**	*5.00*	**0.186**	*5.00*	**0.013**	*5.00*	**0.023**	*5.00*	**0.07** ± 0.08	*5.00 ± 0.00*
					0.125	**0.006**	*5.00*	**0.190**	*5.00*	**0.008**	*5.00*	**0.030**	*5.00*	**0.08** ± 0.09	*5.00 ± 0.00*

Agents Tx1	Agents Tx2	Mechanism Tx1	Mechanism Tx2	Conc (ng/ml) Tx1	Conc (ng/ml) Tx2	RD FA	RD CI	SMS-CTR FA	SMS-CTR CI	RH30 FA	RH30 CI	RH41 FA	RH41 CI	Average FA	Average CI
Vinorelbine	4HC	Microtubule	Alkylator	4	5000	**0.839**	*3.78*	**0.992**	*0.38*	**0.999**	*0.97*	**0.997**	*1.00*	**1.00** ± 0.08	*0.78 ± 1.53*
					2500	**0.583**	*5.00*	**0.992**	*0.24*	**0.831**	*1.09*	**0.889**	*1.45*	**0.90** ± 0.17	*0.93 ± 2.10*
					1250	**0.636**	*4.68*	**0.771**	*3.25*	**0.503**	*0.85*	**0.470**	*5.00*	**0.58** ± 0.14	*3.03 ± 1.89*
					625	**0.639**	*4.24*	**0.754**	*3.11*	**0.400**	*0.72*	**0.551**	*3.78*	**0.57** ± 0.15	*2.54 ± 1.57*
					313	**0.631**	*4.17*	**0.792**	*2.37*	**0.363**	*0.66*	**0.609**	*2.90*	**0.59** ± 0.18	*1.98 ± 1.45*
				2	5000	**0.893**	*2.63*	**0.999**	*0.10*	**0.999**	*0.94*	**0.998**	*0.96*	**1.00** ± 0.05	*0.67 ± 1.06*
					2500	**0.483**	*5.00*	**0.999**	*0.06*	**0.814**	*1.11*	**0.801**	*1.55*	**0.87** ± 0.21	*0.91 ± 2.14*
					1250	**0.356**	*5.00*	**0.639**	*3.43*	**0.377**	*0.97*	**0.201**	*5.00*	**0.41** ± 0.18	*3.13 ± 1.90*
					625	**0.186**	*5.00*	**0.554**	*3.88*	**0.165**	*2.54*	**0.184**	*5.00*	**0.30** ± 0.19	*3.81 ± 1.17*
					313	**0.154**	*5.00*	**0.531**	*3.81*	**0.154**	*2.71*	**0.145**	*5.00*	**0.28** ± 0.19	*3.84 ± 1.10*
				1	5000	**0.870**	*2.59*	**0.999**	*0.09*	**0.999**	*0.94*	**0.998**	*0.98*	**1.00** ± 0.06	*0.67 ± 1.04*
					2500	**0.405**	*4.41*	**0.994**	*0.15*	**0.789**	*1.14*	**0.826**	*1.21*	**0.87** ± 0.25	*0.83 ± 1.85*
					1250	**0.282**	*5.00*	**0.649**	*2.24*	**0.365**	*0.88*	**0.098**	*5.00*	**0.37** ± 0.23	*2.71 ± 2.06*
					625	**0.080**	*5.00*	**0.495**	*2.83*	**0.132**	*2.24*	**0.101**	*5.00*	**0.24** ± 0.20	*3.36 ± 1.44*
					313	**0.053**	*5.00*	**0.286**	*5.00*	**0.099**	*3.68*	**0.086**	*5.00*	**0.16** ± 0.11	*4.56 ± 0.66*
				0.5	5000	**0.867**	*2.47*	**0.999**	*0.09*	**0.999**	*0.94*	**0.998**	*0.97*	**1.00** ± 0.07	*0.67 ± 0.99*
					2500	**0.376**	*3.40*	**0.998**	*0.05*	**0.815**	*1.11*	**0.832**	*1.09*	**0.88** ± 0.27	*0.75 ± 1.41*
					1250	**0.264**	*3.46*	**0.549**	*2.31*	**0.311**	*0.89*	**0.093**	*5.00*	**0.32** ± 0.19	*2.73 ± 1.74*
					625	**0.059**	*5.00*	**0.472**	*1.99*	**0.087**	*2.83*	**0.100**	*5.00*	**0.22** ± 0.20	*3.27 ± 1.53*
					313	**0.011**	*5.00*	**0.215**	*4.22*	**0.063**	*5.00*	**0.061**	*5.00*	**0.11** ± 0.09	*4.74 ± 0.39*
				0.25	5000	**0.806**	*2.69*	**0.999**	*0.09*	**0.999**	*0.94*	**0.998**	*0.98*	**1.00** ± 0.10	*0.67 ± 1.09*
					2500	**0.364**	*2.75*	**0.998**	*0.06*	**0.837**	*1.08*	**0.811**	*1.06*	**0.88** ± 0.27	*0.73 ± 1.12*
					1250	**0.258**	*2.34*	**0.563**	*1.83*	**0.324**	*0.83*	**0.103**	*3.12*	**0.33** ± 0.19	*1.93 ± 0.96*
					625	**0.084**	*5.00*	**0.353**	*2.11*	**0.067**	*2.62*	**0.054**	*4.97*	**0.16** ± 0.14	*3.23 ± 1.53*
					313	**0.035**	*5.00*	**0.179**	*3.14*	**0.041**	*5.00*	**0.056**	*4.50*	**0.09** ± 0.07	*4.21 ± 0.88*
Vinorelbine	Etoposide	Microtubule	Topo II	4	5000	**0.688**	*5.00*	**0.610**	*5.00*	**0.870**	*2.97*	**0.894**	*2.34*	**0.79** ± 0.14	*3.44 ± 1.38*
					1250	**0.507**	*5.00*	**0.656**	*4.47*	**0.577**	*3.04*	**0.501**	*5.00*	**0.58** ± 0.07	*4.17 ± 0.93*
					313	**0.553**	*5.00*	**0.777**	*2.40*	**0.206**	*5.00*	**0.438**	*5.00*	**0.47** ± 0.24	*4.13 ± 1.30*
					78.3	**0.612**	*4.39*	**0.808**	*2.01*	**0.227**	*2.55*	**0.574**	*3.39*	**0.54** ± 0.24	*2.65 ± 1.04*
					19.6	**0.647**	*3.73*	**0.846**	*1.56*	**0.229**	*1.99*	**0.559**	*3.43*	**0.54** ± 0.26	*2.33 ± 1.06*
				2	5000	**0.617**	*5.00*	**0.547**	*5.00*	**0.871**	*2.93*	**0.880**	*2.44*	**0.77** ± 0.17	*3.46 ± 1.35*
					1250	**0.455**	*5.00*	**0.467**	*5.00*	**0.591**	*2.86*	**0.439**	*5.00*	**0.50** ± 0.07	*4.29 ± 1.07*
					313	**0.289**	*5.00*	**0.438**	*5.00*	**-0.002**	*5.00*	**0.201**	*5.00*	**0.21** ± 0.18	*5.00 ± 0.00*
					78.3	**0.129**	*5.00*	**0.481**	*4.20*	**0.006**	*5.00*	**0.085**	*5.00*	**0.19** ± 0.21	*4.73 ± 0.40*
					19.6	**0.124**	*5.00*	**0.395**	*5.00*	**0.017**	*5.00*	**0.097**	*5.00*	**0.17** ± 0.16	*5.00 ± 0.00*
				1	5000	**0.610**	*5.00*	**0.544**	*4.49*	**0.858**	*3.23*	**0.885**	*2.17*	**0.76** ± 0.17	*3.30 ± 1.27*
					1250	**0.465**	*4.82*	**0.462**	*3.58*	**0.554**	*3.24*	**0.443**	*5.00*	**0.49** ± 0.05	*3.94 ± 0.88*
					313	**0.252**	*5.00*	**0.255**	*5.00*	**0.038**	*5.00*	**0.166**	*5.00*	**0.15** ± 0.10	*5.00 ± 0.00*
					78.3	**-0.034**	*5.00*	**0.129**	*5.00*	**0.016**	*5.00*	**0.015**	*5.00*	**0.05** ± 0.07	*5.00 ± 0.00*
					19.6	**0.001**	*5.00*	**0.157**	*5.00*	**0.022**	*5.00*	**0.010**	*5.00*	**0.06** ± 0.07	*5.00 ± 0.00*
				0.5	5000	**0.622**	*5.00*	**0.539**	*3.81*	**0.872**	*2.92*	**0.873**	*2.38*	**0.76** ± 0.17	*3.04 ± 1.15*
					1250	**0.426**	*4.65*	**0.440**	*2.82*	**0.561**	*3.16*	**0.410**	*5.00*	**0.47** ± 0.07	*3.66 ± 1.08*
					313	**0.196**	*5.00*	**0.174**	*5.00*	**0.004**	*5.00*	**0.131**	*5.00*	**0.10** ± 0.09	*5.00 ± 0.00*
					78.3	**-0.026**	*5.00*	**0.095**	*5.00*	**0.016**	*5.00*	**0.013**	*5.00*	**0.04** ± 0.05	*5.00 ± 0.00*
					19.6	**0.016**	*5.00*	**0.060**	*5.00*	**0.042**	*5.00*	**0.000**	*5.00*	**0.03** ± 0.03	*5.00 ± 0.00*
				0.25	5000	**0.576**	*5.00*	**0.511**	*4.15*	**0.847**	*3.49*	**0.859**	*2.67*	**0.74** ± 0.18	*3.44 ± 0.99*
					1250	**0.412**	*4.37*	**0.400**	*2.94*	**0.553**	*3.24*	**0.398**	*5.00*	**0.45** ± 0.08	*3.73 ± 0.96*
					313	**0.194**	*5.00*	**0.125**	*5.00*	**0.020**	*5.00*	**0.119**	*5.00*	**0.09** ± 0.07	*5.00 ± 0.00*
					78.3	**0.017**	*5.00*	**0.044**	*5.00*	**0.007**	*5.00*	**-0.002**	*5.00*	**0.02** ± 0.02	*5.00 ± 0.00*
					19.6	**-0.048**	*5.00*	**0.048**	*5.00*	**0.019**	*5.00*	**-0.021**	*5.00*	**0.02** ± 0.04	*5.00 ± 0.00*

Supplemental Table S1: Fraction Affected (FA) and Combiation Index (CI) values for all combinations across all cell lines tested.

Detailed Drug Information - Alias, Mechanism, Source, and Concentrations								
Agent	Alias or Trade Name	Target of Inhibition // Mechanism	Vendor	Product #	MW (g/mol)	Concentrations in Synergy (5x5) Experiments		
						Top Conc (ng/ml)	% of Cmax	Dilution Factor
**4HC**	4-Hydroxy Cyclophosphamide	DNA Crosslinking // DNA Damage	US Biological	H9015	**293.1**	5000	72	2.0
**Actinomycin D**	Dactinomycin / Cosmegen®	DNA intercalation // Inhibits RNA/DNA synth	Sigma-Aldrich	A9415	**1255.4**	1.0	1.0	2.0
**AZD1775**	MK1775	WEE1 // CDK1 activation	Selleck	S1525	**500.6**	500	61	2.0
**Bortezomib**	Velcade®	Proteasome	Sequoia	SRP02310b	**384.2**	10	8.0	1.5
**Cabozantinib**	XL184	Tyrosine Kinases (c-Met; VEGFR2)	Selleck	S1119	**501.5**	2000	100	2.0
**Etoposide**	VP-16	Topoisomerase II // DNA Damage	Selleck	S1225	**588.6**	5000	25	4.0
**SN-38**	Irinotecan metabolite	Topoisomerase IB // DNA Damage	Sequoia	SRP02075e	**392.4**	5.0	17	2.0
**Vinorelbine**	Navelbine®	Microtubule polymerization // Anti-mitotic	Selleck	S4269	**1079.1**	4.0	3.0	2.0

Supplemental Table S2: Drug information table

Cell Line Information			
Line	Histology	Source	Commercial Source
**RD**	Embryonal	Gift from Dr. Calvin K. Lee (Moffitt)	ATCC® CCL-136™
**SMS-CTR**	Embryonal	Gift from Dr. Calvin K. Lee (Moffitt)	Not Commercially Available
**RH30**	Alveolar	Children's Oncology Group (COG)	Not Commercially Available
**RH41**	Alveolar	Children's Oncology Group (COG)	Not Commercially Available

Supplemental Table S3: Cell line information table
